# A pre-post pilot study with and without subsidy to promote improved backyard poultry-raising practices to reduce exposure to poultry and poultry feces in rural Bangladesh

**DOI:** 10.1371/journal.pgph.0004512

**Published:** 2026-07-21

**Authors:** Laura H. Kwong, Jesmin Sultana, Elizabeth D. Thomas, Mohammad Rofi Uddin, Shifat Khan, Jennifer Ching, Mahfuza Islam, Aminul Islam, Ireen Sultana Shanta, Nadia Ali Rimi, Mahbubur Rahman, Peter J. Winch, Tarique Md. Nurul Huda

**Affiliations:** 1 Division of Environmental Health Sciences, School of Public Health, University of California, Berkeley, California, United States of America; 2 Environmental Health and WASH, Health System and Population Studies Division, icddr, b, Dhaka, Bangladesh; 3 Department of International Health, Johns Hopkins Bloomberg School of Public Health, Baltimore, Maryland, United States of America; 4 Programme for Emerging Infections, Infectious Disease Division, icddr, b, Dhaka, Bangladesh; 5 Global Health and Migration Unit, Department of Women’s and Children’s Health, Uppsala University, Uppsala, Sweden; 6 Department of Public Health, College of Applied Medical Sciences, Qassim University, Buraydah, Saudi Arabia; Rollins School of Public Health: Emory University School of Public Health, UNITED STATES OF AMERICA

## Abstract

Backyard poultry raising is common in rural Bangladesh and many households keep their poultry inside their houses at night. Compared to keeping poultry outside in sheds at night, this practice likely elevates children’s exposure to poultry and poultry feces and associated enteric pathogens. We conducted a two-arm (subsidy and non-subsidy) pre-post pilot study. Households in both arms received the behavior change communication and counseling intervention. Households in the subsidy arm also received ~23 USD for the construction of an improved poultry shed for nighttime housing. We administered a household survey and spot-check at baseline (February 2020) and then after intervention implementation (January-February 2021) among 37 subsidy and 42 non-subsidy households to determine the impact of the intervention on 1) confining poultry in a shed outside of the house at night, 2) poultry feces management, and 3) hand washing with soap after contacting poultry and poultry feces. A secondary aim was to investigate the effectiveness of a monetary subsidy on encouraging poultry raisers to build an improved poultry night shed. At endline, 58% of all households had an improved poultry shed (87% of subsidy and 33% of non-subsidy households). The percentage of all households confining all of their poultry outside the house the previous night was significantly higher at endline (33%) compared to baseline (2.5%) (prevalence difference: 30 percentage points [pp]; 95% confidence interval: [19, 41]). More households (both arms) had no visible poultry feces piles inside the house compared to baseline (prevalence difference: 26pp [10, 41]). Our intervention effectively encouraged households to build poultry sheds, confine poultry outside of house at night, and maintain an indoor living space free of poultry feces. Future studies should assess if housing all poultry outside the house reduces children’s exposure to poultry feces enough to mitigate health risks associated with poultry ownership.

## Introduction

Backyard poultry raising is common across the globe [1]. In Bangladesh, 80% of rural households raise backyard poultry as a source of food for households and income for women [2]. In this setting, humans live near their poultry: in one study among poultry-raising households in Bangladesh, 98% reported poultry scavenging in the yard, 93% said poultry roam freely inside homes during the day, and 37% reported children touching, carrying, or playing with poultry in the past two weeks [3]. It is also rare for backyard poultry producers to implement biosecurity measures that could limit the introduction and spread of disease among birds and to humans (*e.g.*, providing clean litter, limiting contacts with people and other birds, handwashing with soap) [4,5]. Close contact with poultry and poultry feces increases the risk of *Campylobacter* [6–8], *Salmonella* [8], and *Cryptosporidium* infections [9], avian influenza [2], and other negative health outcomes for children, including diarrhea and poor growth [10,11].

In Bangladesh and elsewhere, many small-scale poultry raisers keep their poultry inside their house at night and confine poultry only intermittently during the day so that they are free to scavenge for food [4,12–15]. Housing poultry inside the house at night has been associated with poor health outcomes among young children, including poor child growth [15] and diarrhea [16,17]. While confining poultry outside the house only during the night may not entirely prevent children from contacting poultry or poultry feces, housing poultry outside may reduce children’s exposure to poultry feces enough to reduce their chance of enteric infections. Lack of financial, material, labor, and other resource constraints, and lack of guidance, can result in poultry being housed inside the dwelling [4,12] or outside in rudimentary structures that lack the requirements needed to support poultry health, such as ventilation and ease of cleaning [4,18].

Confining poultry in improved poultry sheds overnight could also improve poultry health and productivity by protecting birds from weather, theft, and predation [4,19,20]. There is no single definition for an improved poultry shed, but generally they are defined as being outside, having multiple compartments, and natural ventilation [21]. Other recommendations for poultry housing include space, light, and protection [22]. Having multiple compartments aids in keeping poultry of different types and ages separate to avoid competition over food and reduce stress [20]. Adequate ventilation improves air quality inside the shed [23,24] and temperature regulation [25]. Housing for backyard poultry is sometimes elevated off the ground to protect birds from snakes and rodents [26]. An elevated floor can also prevent water from runoff or flooding or high moisture from clay soil from entering the shed, and can facilitate airflow [20]. In addition to supporting poultry health, adequate poultry housing can facilitate concentration of poultry feces to be used for agricultural fertilizer [4]; this may also limit the amount of open poultry feces in the domestic environment. As such, providing guidance and support to backyard poultry raisers on improved poultry housing is important for safe, hygienic, and productive poultry raising [4,19].

Since there is limited research on interventions to reduce young children’s exposure to backyard poultry and poultry feces, we conducted a pre-post pilot study in rural Bangladesh to assess the impact of a behavior change communication (BCC) and counseling intervention (with and without a subsidy) on the nighttime confinement location of poultry, the presence of poultry feces in various household locations, and handwashing practices after contacting poultry and poultry feces.

## Materials and methods

### Ethics statement

The protocol (PR-18087) for this study was approved by the Institutional Review Board of icddr,b. This protocol was not separately reviewed by an animal ethics committee; however, intervention recommendations followed FAO Animal Production and Health guidelines for village/backyard poultry [26,27], and an in-country veterinarian (ISS) oversaw intervention design. Enumerators collected informed written consent from the primary poultry-raiser in the study household at enrollment. This trial did not assess any health outcomes and was therefore not registered as a clinical trial. We have included a statement on inclusivity in our global research in S1 Checklist.

### Setting

We conducted this study in the Fulbaria sub-district of Mymensingh district in north-central Bangladesh. Fulbaria is typical of rural sub-districts in Bangladesh; it contains 13 unions, each with several villages. In each village, there were compounds of one to eight households, clustered around a central, shared courtyard. Poultry are typically owned by individual households and the responsibility of one individual; we defined this person, the primary poultry-raiser, as the household member who owned and cared for the poultry and was the primary decision-maker for the poultry. In rural Bangladesh, primary poultry-raisers are most often female [3].

### Study design

We conducted a two-phase study to explore how to reduce children’s exposure to poultry and poultry feces in rural Bangladesh. In Phase I, we conducted formative research to identify and refine existing local strategies that could separate young children from poultry and poultry feces, the results of which are and will be published in companion papers [12]. In Phase II, we conducted a pre-post pilot study to investigate the effectiveness of a neighborhood-based BCC and counseling intervention, with and without monetary subsidy, to promote nighttime confinement of poultry outside the house and improve poultry feces management and handwashing practices. This paper reports on the pilot study.

The primary objective of the pilot study was to compare all enrolled households’ poultry housing and feces management behaviors before and after the intervention. As a secondary objective, we sought to understand the importance of subsidies in facilitating poultry shed construction. To achieve the secondary objective, we included two arms in the study: households in both the subsidy and non-subsidy arms received the BCC and counseling intervention while only households in the subsidy arm received a monetary subsidy of 2000 BDT (USD ~ 23 at the time of implementation) to pay for approximately half the labor and/or material costs associated with constructing an improved poultry shed. We provided the subsidy only after the household showed that it had a contract with a carpenter, a contract to purchase materials, or had the required materials (which were likely purchased but may have been gathered without purchasing). See the supplemental materials for the TiDeiR-WASH checklist (Table A in S1 Appendix) and the CONSORT checklist (Table B in S1 Appendix).

### Selection of study households and participants

We performed a multi-stage sampling process. First, we selected study unions (the smallest administrative unit in Bangladesh); unions were the unit of randomization to reduce the likelihood that participants in the non-subsidy union would learn about the subsidy offered in the subsidy union. From 13 unions in Fulbaria sub-district, we excluded 6 that were urban (because backyard poultry raising was assumed to be less prevalent), hilly (because most of Bangladesh is flat), or outliers in terms of socio-demographic characteristics (*e.g*., literacy rate, household ownership, population density, male-to-female ratio, sanitation coverage, safe drinking water provision at the household level, and electricity supply). From the eligible unions, we used a random number generator to randomly select two. From each selected union, we selected one village based on convenience (road access and distance from the Fulbaria sub-district center), and poultry-raising practices (nighttime confinement of poultry inside the dwelling). Then, to select study compounds (households that shared a common courtyard), trained enumerators identified each village’s center and went door-to-door in a clockwise direction from the center to identify compounds in which: 1) each of the households had at least one adult chicken (at least two months old); 2) at least one household housed poultry inside their dwelling at night; and 3) at least one household had a child 6–59 months old. We excluded compounds with households that engaged in commercial poultry farming (50 + birds) and compounds with a single household not adjacent to other compounds. We enrolled households in neighboring compounds to create a supportive environment for adopting recommended behaviors and creating new poultry-raising norms. The enrollment process was completed in February 2020.

### Outcomes and sample size

The primary outcome was the percentage of households that reported confining all poultry outside the household living space at night. Secondary outcomes included construction and use of improved poultry sheds, the presence of poultry feces in various household locations (*e.g.*, inside the dwelling, courtyard), the presence of a specific pit for disposal of poultry feces, the presence of a handwashing station with soap, and self-reported handwashing after contact with poultry, poultry products (*i.e.,* eggs), or poultry feces. While the primary aim of the study was to investigate the effectiveness of the BCC and counseling intervention on the primary and secondary outcomes, a secondary aim was to investigate the effect of a monetary subsidy on encouraging poultry raisers to build an improved poultry shed for nighttime housing and the study’s primary and other secondary outcomes.

Prior pilot studies suggest that the inclusion of 50 households may be sufficient for assessing the effectiveness of an intervention that aims to increase uptake of technologies and behaviors similar to those in our intervention [28,29]; since we aimed to understand uptake of the night shed with and without a subsidy, we ideally would have enrolled two arms of fifty households each. However, due to budgetary constraints, we were limited to 40 households per arm and enrolled 80 total. Assuming a baseline of 20% of households confining all of their poultry outside at night [2], a sample size of 80 households allowed this pilot study to have a minimum detectable effect of 13 percentage points with 80% power.

### Intervention design

We conducted extensive formative research to develop the intervention. First, we conducted transect walks and informal interviews across Bangladesh to record in photographs and narrative the behaviors and technologies used by backyard poultry-raising households to assist with any of the following: confining poultry during day, housing poultry at night [12], restricting children’s access to poultry and poultry feces, handwashing after handling poultry, and removing poultry feces from the household premise. We then conducted exploratory interviews to understand the feasibility, acceptability, effectiveness, difficulties, benefits, and costs associated with the behaviors and technologies recorded, and held focus group discussions with this same population to further explore backyard poultry management and preferences for intervention delivery. Finally, we conducted a trial of improved practices (TIPS) to evaluate the acceptability and feasibility of poultry confinement and feces management candidate interventions. Detailed descriptions of this formative work are beyond the scope of this manuscript and available elsewhere [12,30]. In brief, we found that there was a preference for an intervention focused on poultry (*e.g.*, poultry confinement) rather than on children (*e.g.*, safe child play spaces). We also found that daytime confinement of poultry was neither acceptable nor feasible, whereas nighttime confinement in a shed separate from the dwelling was both acceptable and feasible, as were recommendations around poultry feces management and handwashing with soap.

Based on findings from this formative research and the literature, we identified four key behavioral recommendations to target in an intervention: 1) confine all poultry out of the house at night; 2) wash hands with soap and water after contact with poultry, poultry products, and poultry feces; 3) remove uncontained poultry feces from indoor and outdoor spaces as soon as you see them; and 4) dispose of poultry feces in a specific place away from children’s reach (*e.g.,* a covered pit). We then listed determinants of these target behaviors as identified through the formative research (*e.g.*, motives and constraints to each behavior) and categorized them according to the levels (societal, community, household, individual and habitual) and dimensions (contextual, psychosocial, and technological factors) of the Integrated Behavioral Model for WASH (IBM-WASH) [31]. We then proposed intervention components for determinants of each target behavior, including BCC content and an enabling technology. Each target behavior had a corresponding recommended enabling technology.

**Fig 1:**
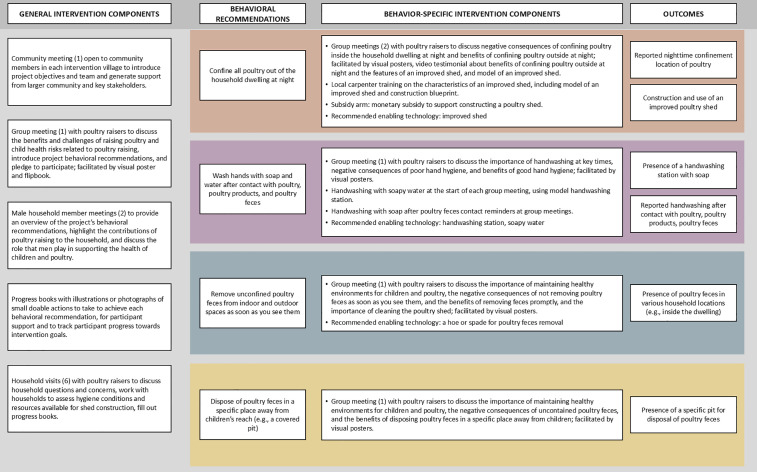
Intervention components, behavioral recommendations, and outcomes.

To deliver the intervention, we developed an approach called Neighborhood-based Environmental Assessment and Planning (NEAP). NEAP is a novel participatory approach that incorporates principles of household-based assessments and an ecological perspective, adapted from an intervention delivered in Bangladesh at the household-level [32]. The intervention was designed to provide recommendations for behaviors and enabling technology to support backyard poultry raisers to adopt improved poultry housing and hygiene practices, with the aim of reducing young children’s exposure to poultry and poultry feces while supporting the health and productivity of the household’s flock. Intervention components are further described below and in Fig 1. The intervention was delivered over three months.

### Intervention delivery – Neighborhood-based Environmental Assessment and Planning (NEAP)

**Fig 2:**
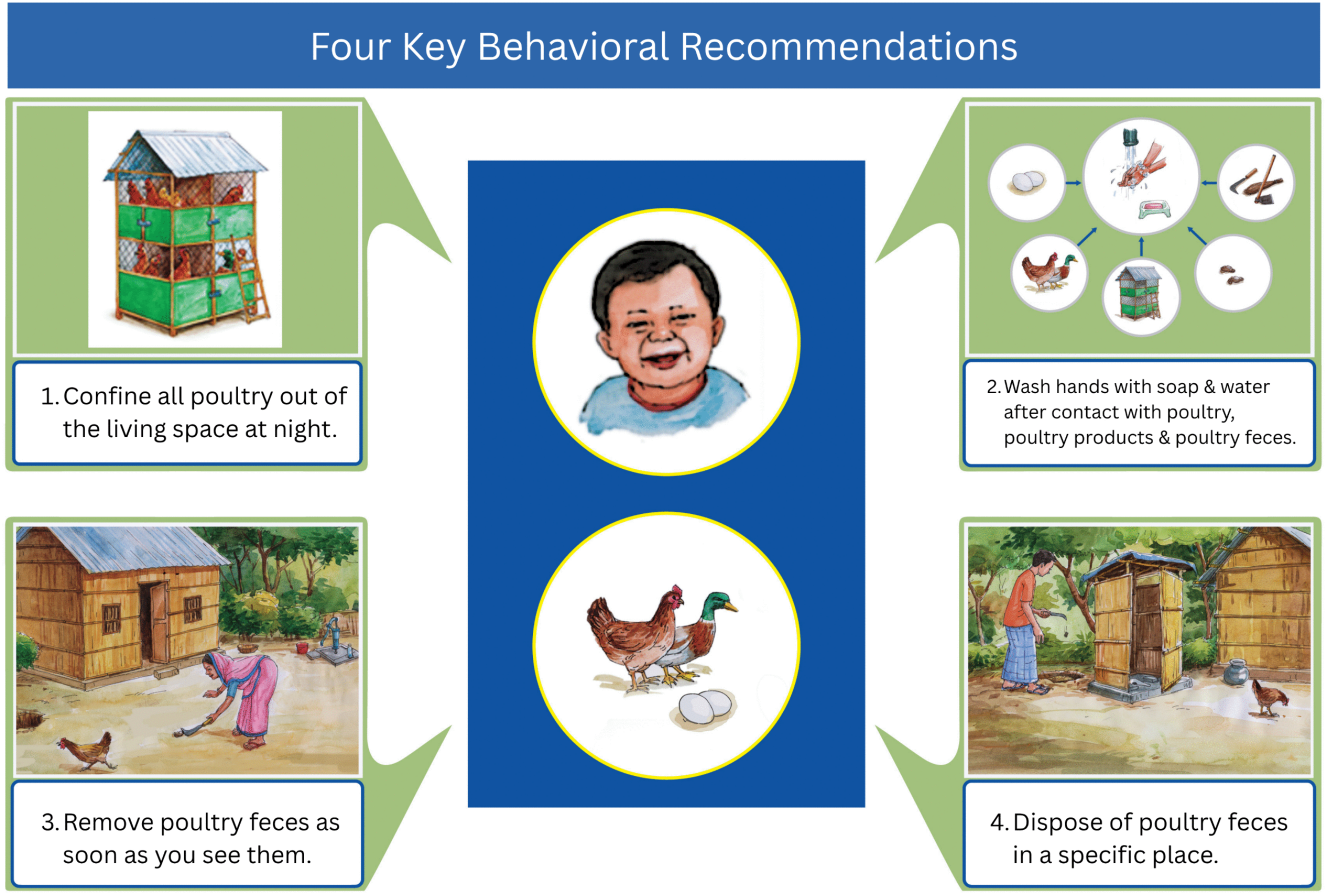
Poster displaying recommended behaviors, shown and discussed at group meetings.

The NEAP approach involves both group meetings and household visits for environmental assessment and problem-solving (see Text B in S1 Appendix for an outline of the NEAP curriculum). To deliver the intervention, we formed neighborhoods, *i.e.,* groups of adjacent households. The primary motivator for creating neighborhoods was that although some behavioral recommendations were framed as a household goal (*e.g.*, building improved poultry shed), others (*e.g.*, disposal of poultry feces) could involve common spaces and were framed as neighborhood goals. We recruited community hygiene promoters (CHPs) from study villages to deliver the intervention; CHPs were >18 years old, had at least eight years of formal education, received rigorous training on the intervention, and were supervised by study staff during group meetings. Intervention delivery included the following components:

**Group meetings for primary poultry-raisers**. Following a community meeting in each village to explain the project, female CHPs held a series of six meetings with groups of primary poultry-raisers from enrolled households, all of whom were women. There were 3 groups per village and 6–8 households per group. The first group meeting was an introductory meeting, and the subsequent five were focused on one or two of the behavioral recommendations. In general, at group meetings, CHPs presented a series of illustrative posters for each behavioral recommendation (Fig 2). The first poster (Fig A in S1 Appendix) depicted illustrations of common poultry-raising behaviors or contexts with negative consequences, based on formative research findings and the literature (*e.g.*, housing poultry inside the dwelling at night and having a snake come into the dwelling to prey on the poultry). In the second poster (Fig B in S1 Appendix), photographs of recommended behaviors with a positive outcome were shown (*e.g.*, behavior: confining all poultry out of the house in an improved shed, outcome: happy household members). The third poster (Fig C in S1 Appendix) presented the enabling technology to facilitate the adoption of the recommended behaviors (*e.g.*, an improved shed). CHPs used the posters as visual aids to facilitate discussions during group sessions. The first poster served to facilitate discussion around common poultry-raising behaviors or contexts that could increase both children’s exposure to poultry and health risks for poultry. Part of this discussion included asking participants to reflect on if their own household or compound experienced the behavior or context depicted on the poster. The purposes of the second and third posters were to facilitate discussions on how to address each situation depicted in the first poster and show target behaviors and outcomes. Prior research has found that in rural Bangladesh, participants responded more positively to illustrations of negative behaviors and photographs of recommended behaviors [32].

In addition to the behavior-specific posters, an overview poster with illustrations of all four key behavioral recommendations was provided to households at the first group meeting. The collective goal of the target behaviors was to have healthy children and healthy poultry; this goal was stated on the overview poster. We asked participants to sign the poster and display them in their homes as a sign of commitment to the recommended behaviors.

To support adoption of the recommendation around handwashing with soap, all group meetings started with participants washing their hands with soapy water, using a model handwashing station.

**Recommended enabling technology.** To support each behavior, we recommended enabling technology, including: 1) an improved shed for confining poultry at night, defined as an outdoor shed with multiple compartments, cross-ventilation, and elevated from the ground; 2) a handwashing station (a bucket with lid and tap, and basin to catch water) and soapy water bottle (a plastic bottle filled with water mixed with detergent powder) to facilitate handwashing after contact with poultry and poultry products; and 3) a hoe or spade for poultry feces removal and disposal (Fig 2). A scale model of the improved shed and demonstration handwashing stations and soapy water bottles were presented during group meetings.

**Fig 3:**
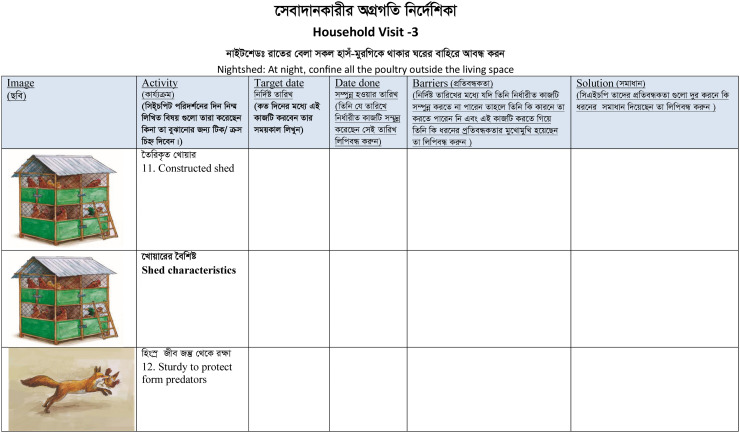
Progress book page.

**Progress books.** To support and track the progress toward each recommended behavior, participants were provided with a progress book with illustrations or photographs of small doable actions to take to achieve each recommendation (Fig 3). CHPs had a separate, similar pictorial progress book to help track participants’ progress and to support ongoing monitoring and tailoring of the intervention.

**Household visits.** After group meetings, a female CHP visited each household (6 planned visits). During household visits, CHPs discussed participants’ questions and concerns, worked with them to assess their household hygiene conditions and resources available for shed construction, and recorded the household’s progress and challenges toward adopting the previous group meeting’s recommended behaviors in their progress books. CHPs also reviewed participants’ progress books, and shared possible solutions to any difficulties reported.

**Male household member engagement meetings.** Male members of enrolled households were invited to participate in separate group meetings, conducted by male CHPs. During these meetings, CHPs provided an overview of the behavioral recommendations, highlighted the important contributions of poultry raising to the household, and emphasized the critical role that men play in achieving the goal of having both healthy children and healthy poultry. Separate meetings for men were included as part of the intervention due to formative research findings that men often play a determinant role in decisions about poultry housing type and location, as well as providing the physical, financial, and/or other resources required for the construction of poultry sheds [12].

**Carpenter training.** We also trained local carpenters in each study village on the characteristics of an improved shed so that they could build improved sheds for study participants (and others) who sought them. Carpenters were provided with a blueprint of an improved shed but were encouraged to work with poultry raisers to design a shed that met their needs. Enrolled households were provided with contact information for trained carpenters but were not obligated to contract them to build a shed.

Due to the COVID-19 pandemic, we delayed implementation of intervention activities until mid-September 2020. The intervention continued until mid-December 2020. To mitigate the risk of COVID-19 transmission during the study, CHPs and participants held group meetings outdoors, wore face masks, and individuals stayed 1 m from each other whenever possible. Before each meeting, both as part of the intervention and to follow COVID-19 handwashing recommendations, CHPs and participants washed their hands using the promoted handwashing station and soapy water bottle.

### Data collection

Separate teams conducted intervention delivery and data collection. Data collection staff (one male and one female) were blinded to the village’s assignment to either the subsidy or non-subsidy arm.

**Household survey.** At baseline (February 2020) and endline (January-February 2021) enumerators administered a structured survey to the primary poultry-raiser in each enrolled household. The survey included questions on demographics and socioeconomic status (baseline only), self-reported poultry-raising practices (*i.e*., day and nighttime poultry confinement (primary outcome), poultry feces management practices (secondary outcome), the involvement of other household members in poultry-raising, and decision-making related to poultry and poultry products, poultry health, observations of children’s interactions with poultry and poultry feces, and handwashing practices (secondary outcome).

**Household spot-checks.** During baseline and endline quantitative assessments, enumerators conducted spot-checks prior to the survey in all study households. To collect data on the number of poultry feces in household spaces (secondary outcome), enumerators visited different locations in the household and compound (*e.g.*, house, courtyard, veranda, and other places) to record signs of poultry (*e.g.*, feathers) and to count visible poultry feces piles. Enumerators counted each pile of poultry feces in each location up to 25 piles and recorded a categorical “>25 piles” if more than 25 piles were present. To assess secondary outcomes related to feces management and handwashing behaviors, enumerators recorded the presence of a specific pit for disposing of poultry feces away from children’s reach and the covering status of the pit, as well as the presence of a handwashing station with soap and water. They also recorded the presence of free-roaming poultry in the household.

Enumerators also recorded the presence of different types of poultry housing (*e.g.*, shed, barricade, bamboo cage, corral, and other confinement strategies) for assessment of whether the household had access to and/or ownership of an improved poultry night shed (secondary outcome). A “shed” was defined as a house-like poultry confinement structure with features including walls, roof, and door. An improved shed was defined as a shed with multiple compartments, ventilation, and an elevated floor. A barricade was poultry housing where various materials were used in conjunction with household furniture or walls to enclose poultry inside the house or a separate building [12]. Bamboo cages were also commonly used to enclose poultry inside dwellings or other buildings. To help differentiate between housing types, structural elements (*e.g.*, windows, ventilation) and signs of use (*e.g.*, poultry feces piles) were recorded. Photographs of poultry housing were also taken.

**Process monitoring and documentation.** We documented intervention activities and CHP performance throughout implementation (see Text A and Table C in S1 Appendix for more detail). For each group meeting and household visit, CHPs recorded meeting attendance, duration, and difficulties related to intervention delivery in a semi-structured daily record form. icddr,b supervisors also observed at least one fortnightly group meeting and two household visits facilitated by each CHP to assess performance, recording their observations in the supervisor monitoring form (see Table D in S1 Appendix for results).

### Data analysis

We summarized socio-demographic characteristics and animal ownership across the intervention arms at baseline. To assess the impact of the intervention, we assessed the change in primary and secondary outcomes from baseline to endline for both study arms combined. To assess the impact of the subsidy on the intervention, we assessed the difference in the change in primary and secondary outcomes from baseline to endline between study arms. Since there were only two villages per arm, we assessed differences between the arms with difference-in-difference analysis. We conducted between-arm analyses according to the arm assignment at enrollment without considering session attendance. We also analyzed results considering whether the household had a poultry shed at endline to assess the association between having a poultry shed and poultry management practices. We report only statistically significant changes and/or differences. There was no missing data. T-tests were conducted using Stata-13 (StataCorp; College Station, TX).

## Results

In February 2020, we enrolled 80 households from four villages of two unions in the Fulbaria sub-district of Mymensingh district. At the endline visit (January-February 2021), one household had migrated out of the study area, resulting in endline data collection for 79 households (Fig D in S1 Appendix). At baseline, most characteristics of primary poultry-raisers and their households were similar across the two arms (Table 1). All the primary poultry-raisers were female. In the non-subsidy arm, access to an improved latrine and ownership of a smartphone was more common, and ownership of bulls/milk cows/buffaloes, a television, and a mobile phone were less common than in the subsidy arm.

**Table 1:** Characteristics of the study population at baseline (February 2020).

Indicators	Non-subsidy arm (n = 42) n (%) or mean ± SD	Subsidy arm (n = 38) n (%) or mean ± SD	Total (n = 80) n (%) or mean ± SD
Status of the respondent			
Female	42 (100%)	38 (100%)	80 (100%)
Has child 6–59 months of age	21 (50%)	22 (58%)	43 (54%)
Average number of individuals in the household	4.0 ± 1.4	4.3 ± 1.4	4.1 ± 1.4
Average number of households in the compound	3.9 ± 1.6	3.0 ± 1.1	3.5 ± 1.4
Survey respondent’s mean age (years)	37 ± 14	35 ± 12	36 ± 13
Survey respondent’s formal education (years)			
No formal education	18 (43%)	16 (42%)	34 (43%)
1–5 years	15 (36%)	13 (34%)	28 (35%)
6–10 years	8 (19%)	9 (24%)	17 (21%)
More than 10 years	1 (2%)	0 (0%)	1 (1%)
Primary occupation of the main earning member of the household (top 6)			
Day/unskilled laborer (domestic, agricultural, or migrant work within the country)	14 (33%)	10 (26%)	24 (30%)
Work on own farm or as a sharecropper	10 (24%)	9 (24%)	19 (24%)
Rickshaw/Van puller/Boat driver	9 (21%)	2 (5%)	11 (14%)
Skilled worker other than carpenter/carpenter (long term contracted laborer)	0 (0%)	7 (18%)	7 (9%)
Business owner	4 (10%)	3 (8%)	7 (9%)
Carpenter/carpenter	1 (2%)	3 (8%)	4(5%)
**Has access to an improved latrine** ^**a**^	**28 (67%)**	**21 (55%)**	49 (61%)
Household assets			
Has electricity (including solar)	42 (100%)	36 (95%)	78 (98%)
**Television (functional)**	**2 (5%)**	**8 (21%)**	10 (13%)
Refrigerator (functional)	1 (2%)	3 (8%)	4 (5%)
**Mobile phone**	**37 (88%)**	**36 (95%)**	73 (91%)
**Smartphone**	**15 (36%)**	**6 (16%)**	21 (26%)
Household poultry ownership			
Chickens (any age)	42 (100%)	38 (100%)	80 (100%)
Average number of chickens (any age) per household	8.8 ± 7.0	8.5 ± 7.9	8.6 ± 7.4
Healthy^b^ adult chickens (>2 months age)	36 (86%)	35 (92%)	71 (89%)
Sick^c^ adult chickens (>2 months age)	0 (0%)	2 (5%)	2 (3%)
Egg-laying chickens	21 (50%)	21 (55%)	42 (53%)
Healthy chicks (<2 months age)	27 (64%)	19 (50%)	46 (58%)
Sick chicks (<2 months age)	6 (14%)	4 (11%)	10 (13%)
Ducks (any age)	9 (21%)	24 (63%)	33 (41%)
Average number of ducks (any age) per household	0.5 ± 1.1	3.0 ± 3.8	1.7 ± 3.0
Household ruminant ownership			
Pigeons	0 (0%)	4 (11%)	4 (5%)
**Bulls/milk cows/buffaloes**	**11 (26%)**	**23 (61%)**	34 (43%)
Goats/sheep	12 (29%)	13 (34%)	25 (31%)

Bolded values indicate those that are significantly different between groups.

^a^Improved toilet according to JMP: Flush or pour-flush to - piped sewer system, septic tank, pit toilet, ventilated improved pit (VIP) toilet, pit toilet with slab, composting toilet, pit latrine with slab, no bucket and/or hanging toilet.

^b^Self-reported by the respondent: Poultry (adult chicken/chick/adult duck/duckling) with no sign symptom of a disease.

^c^Self-reported by the respondent: Poultry (adult chicken/chick/adult duck/duckling) with sign symptom of a disease *(e.g.,* fever, convulsion, pox, diarrhea, vomiting).

On average, 93% of primary poultry-raisers in both arms attended any group meeting. Across arms, 70% (n = 56) of primary poultry-raisers attended all six meetings and 93% (n = 74) attended at least four meetings. For the male engagement meetings, attendance at the first meeting was 89% in the subsidy arm and 85% in the non-subsidy arm but dropped to 76% in the subsidy arm and 39% in the non-subsidy arm for the second male meeting.

### Association between the intervention and poultry sheds, poultry confinement, poultry feces management, and handwashing

The intervention was associated with a higher prevalence of confining all poultry outside the house at night and increased access to any shed and an improved shed (Table 2). At baseline, 2.5% of households reported confining all of their poultry outside their living space at night; this increased substantially by endline (prevalence difference (PD): 30 percentage points [pp], 95% confidence interval (CI): [19, 41]). At baseline, an unimproved shed was present in 38% of households and 0% had an improved shed. The percentage of households that had access to any shed (improved or not) was higher at endline (PD: 33pp [21, 45]); most of the new sheds were improved sheds (PD: 58pp [47, 69]). At endline, sheds were an average of 15 steps (SD: 7 steps) from the front door of the household.

**Table 2:** Impacts of the Neighborhood-based Environmental Assessment and Planning intervention to reduce the prevalence of poultry sleeping indoors at night and reduce the presence of poultry fecal matter in the household environment.

Indicators	Baseline (n = 79) n (%)	Endline (n = 79) n (%)	Prevalence difference (baseline- to- endline) pp [95% CI]
Percent of primary poultry-raisers who self-reported that	n = 79	n = 77	
all poultry were confined outside the living space at night	2 (3%)	25 (33%)	**30 [19, 41]**
Percent of households with observed poultry housing	n = 79	n = 79	
**Access to/ ownership of any shed**	30 (38%)	56 (71%)	**33 [21, 45]**
**Access to an improved**^**a**^ **shed**	0 (0%)	46 (58%)	**58 [47, 69]**
**Ownership of an improved shed**^**b**^	0 (0%)	43 (54%)	**54 [43, 66]**
Uncontained poultry feces piles observed inside the house	n = 79	n = 78	
**No feces pile**	43 (54%)	63 (81%)	**26 [10, 41]**
**1–25 feces piles**	33 (42%)	13 (17%)	**-26 [-40, -11]**
> 25 feces piles	3 (4%)	2 (3%)	0 [0, 0]
Uncontained poultry feces piles observed in the veranda	n = 60	n = 61	
No feces pile	20 (33%)	24 (39%)	5 [-12, 23]
1–25 feces piles	35 (58%)	33 (54%)	-3 [-21, 14]
> 25 feces pile	5 (8%)	4 (7%)	-2 [-5, 2]
Uncontained poultry feces piles observed in the courtyard^c^	n = 79	n = 77	
No feces pile	0 (0%)	1 (1.3%)	1 [-1, 4]
1–25 feces piles	12 (15%)	10 (13%)	-3 [-13, 8]
> 25 feces pile	67 (85%)	66 (86%)	1 [-9, 12]
Poultry feces disposal sites (spot-check confirmed)	n = 79	n = 79	
**Specific place**	16 (20%)	51 (65%)	**44 [31, 58]**
**Used as a fertilizer in crop field or garden**	37 (47%)	16 (20%)	**-27 [-39, -14]**
**Bush or jungle**	28 (35%)	5 (6%)	**-29 [-41, -17]**
**Drain or ditch**	14 (18%)	3 (4%)	**-14 [-23, -5]**
Area beyond the courtyard	1 (1%)	6 (8%)	6 [0, 13]
Water bodies (pond/lake)	0 (0%)	1 (1%)	1 [-1, 4]
Percent of households with observed access to a handwashing station with water and soap/soapy water	n = 79 28 (35%)	n = 79 36 (46%)	10 [-5, 25]
Percent of primary poultry-raisers who self-reported washing hands with soap and water….	n = 78	n = 79	
After defecation	60 (77%)	52 (66%)	-10 [-23, 3]
After cleaning child feces	7 (9%)	9 (11%)	3 [-6, 11]
**Before eating**	16 (21%)	38 (48%)	**28 [14, 42]**
**Before serving food**	0 (0%)	3 (4%)	4 [0, 8]
**Before preparing food**	4 (5%)	47 (60%)	**54 [43, 66]**
**After handling poultry feces**	11 (14%)	51 (65%)	**51 [37, 64]**
**After feeding poultry/ handling poultry or poultry products**	1 (1%)	35 (44%)	**43 [31, 55]**
**After handling other animal feces**	15 (19%)	27 (34%)	**15 [4, 26]**

Bolded values indicate those that are significantly different between timepoints.

^a^An outdoor, multi-compartment poultry night-shed with cross-ventilation that is elevated off the ground.

^b^Three households had access to an improved shed that were owned by other households of the same compound.

^c^During endline enumerators could not access the courtyards of two non-subsidy households to observe and count the poultry feces.

The intervention increased the percentage of households that were free of uncontained feces indoors, but not outdoors (Table 2). At baseline, 54% of households had no uncontained feces (feces outside of the poultry confinement structure) observed inside the house, which increased to 81% at endline (PD: 26pp [12, 41]). This aligned with the reduction in respondent observation of uncontained poultry feces in the house in the past week (PD: -17pp [-29, -4]). However, over 60% of households had uncontained feces on the veranda and nearly 100% had uncontained feces in the courtyard at both baseline and endline.

With regards to disposal of and contact with poultry feces, the intervention increased disposal of feces in a specific pit/pile or as fertilizer and reduced disposal in the bush or in a drain (Table 2). At baseline, 47% of households used poultry feces as a fertilizer, 35% threw them in the bush, 18% threw them in a drain or ditch, and 20% disposed of them in a specific place such as a trash pile. The percentage of households that disposed of poultry feces in a trash pile or pit increased substantially after the intervention (PD: 44pp [31, 58]). Caregivers also reported a reduced prevalence of contact between their <5 children and poultry feces in the past week (PD: -22pp [-38, -6]) and of their <5 children entering a poultry confinement structure in the past week (PD: -32pp [-52, -12]) (Table E in S1 Appendix).

Additionally, the intervention was associated with increased self-reported handwashing with soap at multiple key times, but no change in access to a handwashing station with soap (Table 2). At baseline, most primary poultry-raisers (77%) reported handwashing with soap after defecation; however, handwashing before eating, after handling poultry feces, and after handling other animal feces was reported by about one-fifth. Fewer than 10% of respondents said they washed hands with soap after cleaning child feces, after feeding poultry/handling poultry or poultry products, or before preparing or serving food. At endline, the prevalence of primary poultry-raisers who self-reported washing their hands with soap increased substantially to 65% after handling poultry feces (PD: 51pp [37, 64]), 44% after feeding poultry/handling poultry or poultry products (PD: 43pp [31, 55]), and 34% after handling other animal feces (PD: 15pp [4, 26]). There was also a substantial increase in the prevalence of self-reported handwashing with soap before preparing food (PD: 54pp [43, 66]) and before eating (PD: 28pp [14, 42]).

The intervention did not result in any adverse or unintended effects.

### Association between the subsidy and poultry sheds, poultry confinement, poultry feces management, and handwashing

The percentage of households that reported confining all of their poultry outside their living space at night increased in both study arms (non-subsidy arm PD: 24pp [11, 38]; subsidy arm PD: 36pp [18, 54]) and there was no difference between arms (difference-in-difference (DID): 12pp [-10, 34]) (Table 3). While the increase in access to improved sheds occurred in both study arms (non-subsidy arm PD: 33pp [18, 48]; subsidy arm PD: 87pp [75, 98]), the increase was higher in the subsidy arm (DID: 53pp [34, 72]). The subsidy was not associated with changes in the percentage of respondents who reported seeing uncontained feces in the house, on the veranda, or in the courtyard (Table F in S1 Appendix).

**Table 3:** Impacts of the Neighborhood-based Environmental Assessment and Planning intervention to reduce the prevalence of poultry sleeping indoors at night and reduce the presence of poultry fecal matter in the household environment, by study arm.

Indicators	Baseline n (%)		Endline n (%)		Prevalence Difference (baseline- to- endline)		Effect size (difference-in-differences) pp (95% CI)
Non-subsidy	Subsidy	Non-subsidy	Subsidy	Non-subsidy pp (95% CI)	Subsidy pp (95% CI)
Percent of households reported confined all poultry outside the living space at night	n = 42	n = 37	n = 41	n = 36	n = 41	n = 36	
	1 (2%)	1 (3%)	11 (27%)	14 (39%)	**24 [11, 38]**	**36 [18, 54]**	12pp [-10, 34]
Poultry housing	n = 42	n = 37	n = 42	n = 37	n = 42	n = 37	
**Access to/ ownership of any shed**	15 (36%)	15 (41%)	23 (55%)	33 (89%)	**19 [2, 36]**	**49 [32, 66]**	**30 [6, 53]**
**Access to an improved**^**a**^ **shed**	0 (0%)	0 (0%)	14 (33%)	32 (87%)	**33 [18, 48]**	**86 [75, 98]**	**53 [34, 72]**
**Ownership of an improved shed**^**b**^	0 (0%)	0 (0%)	12 (29%)	31 (84%)	**29 [14, 43]**	**84 [71, 96]**	**55 [36, 74]**
Uncontained poultry feces piles observed in the house	n = 42	n = 37	n = 41	n = 37	n = 41	n = 37	
No feces pile	21 (50%)	22 (60%)	35 (85%)	28 (76%)	**34 [15,54]**	16 [-8, 40]	-18 [-48, 12]
1–25 feces piles	19 (45%)	14 (38%)	5 (12%)	8 (22%)	**-34 [-54, -15]**	-16 [-39, 7]	18 [-11, 47]
> 25 feces piles	2 (5%)	1 (3%)	1 (2%)	1 (3%)	0 [-7, 7]	0 [-8, 8]	0 [-10, 10]
Uncontained poultry feces piles observed in the veranda	n = 35	n = 25	n = 35	n = 26	n = 33	n = 25	
No feces pile	15 (43%)	5 (20%)	16 (46%)	8 (31%)	3 [-24, 30]	8 [-12, 28]	5 [-30, 40]
1–25 feces piles	20 (57%)	15 (60%)	19 (54%)	14 (54%)	-3 [-30, 24]	-4 [-26, 18]	-1 [-37, 35]
> 25 feces piles	0 (0%)	5 (20%)	0 (0%)	4 (15%)	–	-4 [-12, 4]	-4 [-11, 3]
Uncontained poultry feces piles observed in the courtyard^c^	n = 42	n = 37	n = 40	n = 37	n = 40	n = 37	
No feces pile	0 (0)	0 (0)	0 (0)	1 (3%)	–	3 [-3, 8]	3 [-2, 8]
1–25 feces piles	9 (21%)	3 (8%)	5 (13%)	5 (14%)	-10 [-26, 6]	5 [-8, 19]	15 [-5, 36]
> 25 feces piles	33 (79%)	34 (92%)	35 (88%)	31 (84%)	10 [-6, 26]	-8 [-23, 6]	-18 [-39, 3]
**Percent of households dispose of poultry feces in a specific pit/trash**	14 (33%)	2 (5%)	26 (62%)	25 (68%)	**29 [8, 50]**	**62 [46, 79]**	**34 [7, 60]**
Percent of households with access to handwashing station with water and soap/soapy water	n = 42 17 (40%)	n = 37 11 (30%)	n = 42 17 (40%)	n = 37 19 (51%)	0 [-21, 21]	22 [-1, 44]	22 [-8, 52]
Percent of primary poultry-raisers reported washing hands with soap and water…	n = 42	n = 36	n = 42	n = 37	n = 42	n = 37	
After defecation	35 (83%)	25 (69%)	33 (79%)	19 (51%)	-5 [-18, 9]	-16 [-39, 7]	-11 [-37, 14]
After cleaning child feces	5 (12%)	2 (6%)	5 (12%)	4 (11%)	0 [-14, 14]	5 [-6, 16]	5 [-12, 23]
Before eating	11 (26%)	5 (14%)	23 (55%)	15 (41%)	**29 [8, 50]**	**27 [7, 47]**	-2 [-30, 27]
Before serving food	0 (0%)	0 (0%)	1 (2%)	2 (5%)	2 [-2, 7]	5 [-2, 13]	3 [-6, 12]
Before preparing food	3 (7%)	1 (3%)	28 (67%)	19 (51%)	**60 [43, 76]**	**49 [32, 66]**	-11 [-35, 13]
After handling poultry feces	5 (12%)	6 (17%)	23 (55%)	28 (76%)	**43 [23, 62]**	**59 [39, 79]**	17 [-11, 44]
After feeding poultry/ handling poultry or poultry products	1 (2%)	0 (0%)	14 (33%)	21 (57%)	**31 [15,47]**	**57 [40, 74]**	**26 [3, 49]**
After handling other animal feces	3 (7%)	12 (33%)	8 (19%)	19 (51%)	**12 [2, 22]**	19 [-2, 39]	7 [-15, 29]

Bolded values indicate those that are significantly different between groups across timepoints.

a An outdoor, multi-compartment poultry night-shed with cross-ventilation that is elevated off the ground.

b Three households had access to an improved shed that were owned by another household in the same compound.

^c^During endline enumerators could not access the courtyards of two non-subsidy households to observe and count the poultry feces.

The increase in the percentage of households that disposed of poultry feces in a specific pile or pit was also higher among subsidy vs. non-subsidy households (DID: 34pp [7, 60]) (Table 3). There were no other differences in poultry feces disposal or <5-year-old children’s reported contact with poultry or poultry feces between arms.

The percentage of primary poultry-raisers who reported washing their hands after feeding/handling poultry or poultry products increased more in the subsidy arm than in the non-subsidy arm (DID: 26pp [3, 49]); no other findings were different between study arms.

### Association between improved poultry sheds and poultry confinement, poultry feces management, and handwashing

Among households with any shed at endline, 48% confined all of their poultry outside the house at night compared to 0% among those who did not have access to any shed (PD: 48pp [34, 62]). Among households with access to an improved poultry shed at endline, 53% confined all of their poultry outside the house at night compared to 3% of those without an improved shed (PD: 50pp [34, 66]). The 47% of households that had an improved poultry shed at endline but did not confine all of their poultry outside the house at night stated that they were concerned about predators (18/47, 38%) and theft (23/47, 49%). The types of poultry these households continued to keep inside their dwelling were egg laying hens (n = 11), adult chickens (n = 9), chicks (n = 8), ducklings (n = 6), and adult ducks (n = 3). At endline, households that confined all poultry outside at night had a higher prevalence of reporting no feces in the house compared to households that did not confine all poultry outside at night (PD: 17pp [1, 33]).

## Discussion

In this study, we pilot tested an intervention to encourage backyard poultry-raising households to confine poultry outside of the house in an improved shed at night and improve poultry feces management and handwashing practices. The intervention was delivered using a novel neighborhood-based intervention (NEAP), which involved all poultry-raising households in shared and adjacent compounds, delivered by village-based CHPs who facilitated meetings with primary poultry-raisers and, separately, their male household members, and conducted household visits for environmental assessment and problem-solving. CHPs and household members tracked progress and difficulties in completing “small doable actions” [33] to achieve the project’s behavioral recommendations. The intervention was successful at encouraging poultry-raising households to build (improved and unimproved) poultry sheds, confine poultry outside of the house at night, and maintain an indoor living space free of poultry feces. Both households that did and did not receive a monetary subsidy constructed improved sheds, with a greater increase among households who received a subsidy to support shed construction. The effectiveness of the NEAP intervention may stem from its social mobilization component, which has also been effective at changing latrine construction and open defecation behaviors in Community-Led Total Sanitation (CLTS) programs [34,35].

### Construction and use of improved poultry sheds

At baseline, housing poultry in a shed overnight was uncommon, with only one-third of households having sheds and only 3% confining all poultry outside at night. The intervention increased the percentage of households with a poultry shed by a notable 33 percentage points. Factors that may have facilitated this high uptake include the neighborhood commitment and check-ins with study staff to help brainstorm solutions to problems as part of NEAP, training on shed construction provided to local carpenters, sessions for male household members that emphasized the importance of their engagement for child and poultry health, and the subsidy that some households received. Few other studies have encouraged households to construct their own poultry sheds. The post-intervention increase in shed ownership in our study was similar to another study in rural Bangladesh, where a homestead intervention provided households with partial reimbursement for the cost of an improved poultry shed [21,36]. In a study in Ethiopia, which required farmers to agree to build a “night shelter, daytime enclosure, or partitions” for poultry received through the intervention, “enclosed coop” ownership increased post-ownership, but by less than in our study (15 percentage points) [14]. The success of both projects in Bangladesh compared to the one in Ethiopia may be because the focus in Bangladesh was on increasing ownership of a shed for nighttime confinement of poultry. Daytime confinement of poultry is not typical for backyard poultry raisers [26], in part due to additional costs for feed that this would necessitate [37]. In addition, during our formative research, we documented widespread use of nighttime poultry sheds in Bangladesh, and preference and willingness to confine poultry separate from the dwelling at night, provided a safe and secure shed was available [12]. Therefore, the success of our intervention may be because it facilitated a widely acceptable and aspirational practice for poultry raisers in this setting. Despite the success of the intervention at increasing shed access and use, some households that had a shed did not use it to confine all of their poultry at night; concerns for protection from predators, weather, and theft, as well as households having multiple types and ages of poultry were the likely contributing factors to this finding. Other studies have also found that even if households have an outdoor confinement structure, they may still choose to keep some or all of their poultry inside the house at night for similar reasons [13,14]. In our formative research, we found that households with sheds would still house egg-laying hens and chicks within their home for short periods of time [12]. To address continued housing of egg-laying hens and chicks inside the dwelling, sheds could include protected compartments for them. In our intervention, we did recommend that sheds have multiple compartments, specifically to address this need. However, building a shed with multiple compartments may have been cost-prohibitive for some participating households. To address concerns about predators, weather, and theft, shed designs could be tailored to be made from stronger, more durable materials, include smaller openings that still provide adequate ventilation without risking predator attacks, include insulation against cold or fans to combat heat, and doors could be fitted with locks; these modifications would come with added costs. Additional behavior change communication may be required to convince households that these improved sheds would address their concerns and an analysis of the return-on-investment of these sheds and more basic sheds may be necessary to justify the additional shed cost.

A setting-dependent issue to consider is the availability of trained carpenters who can build sheds. In our study, we found that households used both study-trained carpenters, carpenters not trained by our study, and male household members who were not carpenters: 38% of households had their shed built by a household member or someone who lives in the same compound, suggesting that finding someone to build the shed may not be difficult in this setting. This finding also confirms the importance of including male household members in the intervention.

### Association between subsidy and shed construction

Our study suggests that while subsidies were helpful for households to construct a shed within a short time period, they may not be necessary for all rural Bangladeshi households to construct poultry sheds. While over >80% of households that received a 23 USD subsidy constructed an improved shed, one-third of households that did not receive a subsidy also constructed an improved shed during the 3-month intervention period. Interviews conducted with participants following the intervention [30,38] indicated that the intervention timeline was too short for some households to arrange the cash, materials, or labor needed for a shed. A few households said that they would build a shed when materials became available, like after a household renovation. Studies that follow up with households 6 or 12 months after the intervention may be better positioned to assess the impact of the intervention on households that do not receive a subsidy.

This intervention may be more scalable if subsidies are not required. While subsidies have the potential to increase equity in sanitation access and use when appropriately and accurately targeted and implemented, past attempts to subsidize sanitation hardware have distorted sanitation markets and created perverse incentives among beneficiaries while primarily benefitting wealthier households and failing to create sustained hardware use [39,40]. In addition, the expectation of a subsidy was noted as a constraint to unsubsidized latrine construction in 15% of CLTS interventions [34]. However, behavior change promotion with a monetary subsidy has been more effective in increasing latrine coverage (or attenuating declines in latrine use) than behavior change promotion alone [41–43], so it is useful to identify how to most cost-effectively implement subsidy programs and evaluating their effect. Some subsidy approaches, such as vouchers and rebates to cover full or partial costs, can be used to deliver subsidies that stimulate both demand and supply, at least in the short term [41–44]. While subsidy validity periods can strongly impact subsidy uptake, vouchers can effectively support households in overcoming liquidity constraints [44]. The optimal amount of the subsidy, which minimizes the implementer’s cost and maximizes uptake, is not clear and likely differs for each household, depending on the total cost of the hardware, the household’s financial status, and the household’s willingness to pay [45]. Appropriately targeting subsidies is difficult as different classification methods identify different households as “poor” [46]. Additionally, households that would benefit from financial subsidies may also need other types of support (such as gender and inclusion initiatives) to harness the long-term benefits of sanitation and other programs [47].

### Poultry feces presence and disposal

The lower prevalence of uncontained feces in the household following the intervention is consistent with prior studies that have found poultry housing associated with less feces in some (but not necessarily all) domestic spaces. Studies in Ethiopia and Uganda suggest that poultry housing is associated with a lower prevalence and/or count of (presumably, uncontained) feces inside the house or outside in domestic spaces, but details on where feces were observed are insufficient to identify why this difference may have been observed [13,14]. In our study, while the reduction in uncontained feces within the home may have been due to poultry spending less time in the home and more time in poultry confinement structures, it was likely also the result of increased cleaning, following the intervention’s recommendation to pick up feces immediately after they are seen. We encouraged primary poultry-raisers to thoroughly scrape animal feces off the ground using a ubiquitously owned garden hoe, with the hope that this would reduce fecal contamination of the underlying and nearby soil. We did not recommend the use of a modified hoe developed specifically for the purposes of animal feces removal [48], which had low uptake when implemented as part of a multi-component water, sanitation, and hygiene intervention in rural Bangladesh despite high uptake of most other intervention components [49].

The percentage of households that had a specific pit for disposing of poultry feces away from children’s reach increased 44 percentage points from baseline to endline. If used consistently, disposing of feces in such a pit may reduce children’s exposure to poultry feces. However, heavy monsoon rains could flood piles or pits containing feces and spread fecal matter across the domestic environment, which may explain the increased rates of environmental contamination and diarrhea observed following heavy rains in this setting [50–52]. While poultry feces could be disposed of in on-site feces containment approaches that are resilient to the weather and climate-sensitive [53], these would not allow for poultry feces to then be used as fertilizer or fish feed.

### Potential for health impact

While it is well-established that children’s contact with poultry and poultry feces is risky for child health, it is not clear that poultry confinement is necessarily beneficial for child health. It appears that the details of how households implement confinement can create benefit, harm, or no effect; our understanding of the impact between poultry housing and child health is also limited by our current indicators. Although children in rural Bangladesh are exposed to enteric pathogens through a number of pathways, principally ingestion of soil contaminated by animal feces [54,55], poultry confinement interventions may still reduce their exposure to poultry and poultry feces enough to reduce diarrhea. For example, in our study context, poultry kept inside the house are often kept under the bed where the child sleeps [2], potentially exposing them to poultry debris or feces particles that become airborne due to poultry movement or sweeping; confinement structures that are used to keep all poultry outside of the house at night could reduce children’s potentially intense indoor exposure at night. Our intervention was also associated with an increase in the percentage of households that had no uncontained feces inside the house. Given that young children in rural Bangladesh are inside about half of their waking hours [56], this reduction in indoor exposure during the night and day could result in long-term health improvements if the behavior change was sustained. Similarly, research on *Campylobacter* transmission in Ethiopia found that infants <13 months old in households that did not keep chickens unconfined at home had lower concentrations of *Campylobacter* in their stool [57]. However, increased confinement may have no effect on child health. In a randomized controlled trial in Ethiopia that distributed chickens and basic husbandry guidance, households in intervention villages were more likely than households in control villages to have a chicken coop, have a coop separated from the house, and have a coop where chickens were confined at both 9 and 18 months after the intervention. However, the intervention had no impact on the 2-week prevalence of diarrhea, vomiting, fever, or anthropometry at endline [58]. Similarly, a Bangladeshi homestead food production program with poultry and food hygiene intervention components increased ownership of poultry sheds, but had no impact on diarrhea prevalence [21,59]. Corralling poultry could also contribute to worse health outcomes, despite a reduced presence of feces indoors, if children enter the poultry shed. While the average distance from the house to the shed was 15 steps (approximately 10 m) in our study, 36% of respondents reported observing their <5-year-old children entering the shed at least once in the past month at endline. In a randomized controlled trial in peri-urban Peru, households corralling chickens doubled the incidence of *Campylobacter-*associated diarrhea for children and in households with >20 chickens, the incidence increased by seven-fold [29]. The authors speculate that these adverse health outcomes were because sheds were built near household entryways, making it easy for children to frequently enter the shed and for environmental factors to contaminate domestic spaces frequented by children; household members that cleaned the sheds may also not have adequately washed their hands afterwards, given water scarcity in the community. Given the range of these findings, further research on the impact of poultry housing variations on children’s exposure to *Campylobacter* and diarrheal outcomes is needed to establish the contexts in which poultry housing is most beneficial, and to determine factors affecting the sustainability of behavior change and outdoor coops.

### Limitations

A primary limitation of this pilot study is that it did not have a control group. As such, the trends we observed could have been due to changes over time rather than the intervention. However, given that there were only 3 months between intervention and follow-up, we do not expect to see such large prevalence differences due to time trends alone. Second, while we conducted spot-checks to gather data on the presence of poultry confinement structures, soap and water at handwashing stations, and feces disposal locations, we relied on self-reported poultry confinement, handwashing, and feces disposal behaviors, and past studies have shown that people tend to over-report behaviors that correspond to the prescriptive norm [60]; they may also have highly inaccurate recall of behaviors that do not seem important, suggesting that direct observation would be helpful to more accurately quantify changes in exposure due to a penning observation [61]. While objective methods could have been used for some of these indicators, such as GPS trackers on poultry to understand where they were confined at night [62], these methods may not have been precise enough and were financially infeasible in this study. Another limitation is that Bangladesh imposed nationwide lockdowns due to COVID-19 following the baseline survey and prior to initiation of the intervention; these lockdowns produced considerable economic hardship [63]. An even greater percentage of households may construct improved poultry sheds in non-pandemic contexts. Additionally, the subsidy may have been used for purposes other than constructing the improved shed, but we did not measure other outcomes that may have been affected. The high number of analytic comparisons increased the possibility that some results were statistically significant by chance alone. Budget constraints limited both the sample size and duration of follow-up, precluding our ability to infer causal mechanisms and assess intervention sustainability and shed durability; a long-term follow-up is now underway.

## Conclusion

The Neighborhood-based Environmental Assessment and Planning (NEAP) intervention successfully encouraged backyard poultry-raising households in rural Bangladesh to construct poultry sheds, confine poultry outside of the house at night, reduce poultry feces in indoor living spaces, and wash hands at some key times. Both households that did and did not receive a monetary subsidy constructed improved sheds. Future research could assess if this intervention and approach is able to create sustained use of poultry night sheds, handwashing at key times, and cleaning of indoor spaces. If so, further work could assess the impact of the intervention on clinical and subclinical *Campylobacter* infections and linear growth among young children.

## Supporting information

S1 Appendix Table A: TiDieR-WASH Checklist. Table B: CONSORT 2010 checklist of information to include when reporting a randomised trial. Text A: Process monitoring. Table C: Summary of process monitoring during an intervention to encourage improved poultry and poultry feces management among households in rural Bangladesh. Table D: Supervisor assessment of community health promoter (CHP) performance during the group meetings to encourage improved poultry and poultry feces management among households in rural Bangladesh. Text B: Curriculum for Neighborhood-based Environmental Assessment and Planning (NEAP) approach to promote nighttime confinement of poultry outside the house and improve poultry feces management and handwashing practices. Fig A: Poster showing some negative consequences of keeping poultry indoors at night, shown and discussed during group meetings to encourage improved poultry and poultry feces management among households in rural Bangladesh. Fig B: Posters showing intended behavior, shown and discussed during group meetings to encourage improved poultry and poultry feces management among households in rural Bangladesh. Fig C: Poster showing the enabling technology of an improved poultry night shed, shown and discussed during group meetings to encourage improved poultry and poultry feces management among households in rural Bangladesh. Fig D: Flow chart of study household selection, enrollment, and participation. Table E: Impacts of the Neighborhood-based Environmental Assessment and Planning intervention to reduce the prevalence of poultry sleeping indoors at night and reduce the presence of poultry fecal matter in the household environment. Table F: Impacts of the Neighborhood-based Environmental Assessment and Planning intervention to reduce the prevalence of poultry sleeping indoors at night and reduce the presence of poultry fecal matter in the household environment, by study arm.
(DOCX)

S1 Checklist Inclusivity in global research. (PDF)
