# Supplementary material for: A pre-post pilot study with and without subsidy to promote improved backyard poultry-raising practices to reduce exposure to poultry and poultry feces in rural Bangladesh
Source: PLOS Glob Public Health. 2026 Jul 21;6(7):e0004512. doi: 10.1371/journal.pgph.0004512 (PMC13387569; doi:10.1371/journal.pgph.0004512)
Supplement: S1 Appendix — Table B: CONSORT 2010 checklist of information to include when reporting a randomised trial. Text A: Process monitoring. Table C: Summary of process monitoring during an intervention to encourage improved poultry and poultry feces management among households in rural Bangladesh. Table D: Supervisor assessment of community health promoter (CHP) performance during the group meetings to encourage improved poultry and poultry feces management among households in rural Bangladesh. Text B: Curriculum for Neighborhood-based Environmental Assessment and Planning (NEAP) approach to promote nighttime confinement of poultry outside the house and improve poultry feces management and handwashing practices. Fig A: Poster showing some negative consequences of keeping poultry indoors at night, shown and discussed during group meetings to encourage improved poultry and poultry feces management among households in rural Bangladesh. Fig B: Posters showing intended behavior, shown and discussed during group meetings to encourage improved poultry and poultry feces management among households in rural Bangladesh. Fig C: Poster showing the enabling technology of an improved poultry night shed, shown and discussed during group meetings to encourage improved poultry and poultry feces management among households in rural Bangladesh. Fig D: Flow chart of study household selection, enrollment, and participation. Table E: Impacts of the Neighborhood-based Environmental Assessment and Planning intervention to reduce the prevalence of poultry sleeping indoors at night and reduce the presence of poultry fecal matter in the household environment. Table F: Impacts of the Neighborhood-based Environmental Assessment and Planning intervention to reduce the prevalence of poultry sleeping indoors at night and reduce the presence of poultry fecal matter in the household environment, by study arm. (DOCX) [file pgph.0004512.s001.docx]

S1 Appendix:

A pilot pre-post trial with and without subsidy to promote safe backyard poultry-raising practices to prevent exposure to poultry and poultry feces in rural Bangladesh

Authors: Laura H. Kwong^1^*, Jesmin Sultana^2^, Elizabeth D. Thomas^3^, Mohammad Rofi Uddin^2^, Shifat Khan^2^, Jennifer Ching^3^, Mahfuza Islam^1,2^, Aminul Islam^2^, Ireen Sultana Shanta^2^, Nadia Ali Rimi^2^, Md. Mahbubur Rahman^2^, Peter J. Winch^3^, Tarique Md. Nurul Huda^2,4^

Affiliations:

^1^ Division of Environmental Health Sciences, School of Public Health, University of California, Berkeley, California, United States of America

^2^ Environmental Health and WASH, Health System and Population Studies Division, icddr,b, Dhaka, Bangladesh

^3^ Department of International Health, Johns Hopkins Bloomberg School of Public Health, Baltimore, Maryland, United States of America

^4^ Department of Public Health, College of Applied Medical Sciences, Qassim University, Buraydah 51452, P.O. Box 6666, Saudi Arabia

*Corresponding author: Laura H Kwong

Email: [lakwong@berkeley.edu](mailto:lakwong@berkeley.edu)

Contents

[Table A: TiDieR-WASH Checklist 4](#_Toc233313576)

[Table B: CONSORT 2010 checklist of information to include when reporting a randomised trial* 1](#_Toc233313577)

[Text A: Process monitoring 1](#_Toc233313578)

[Table C: Summary of process monitoring during an intervention to encourage improved poultry and poultry feces management among households in rural Bangladesh 2](#_Toc233313579)

[Table D: Supervisor assessment of community health promoter (CHP) performance during the group meetings to encourage improved poultry and poultry feces management among households in rural Bangladesh 3](#_Toc233313580)

[Text B: Curriculum for Neighborhood-based Environmental Assessment and Planning (NEAP) approach to promote nighttime confinement of poultry outside the house and improve poultry feces management and handwashing practices 4](#_Toc233313581)

[Session # 5](#_Toc233313582)

[Community meeting 5](#_Toc233313583)

[Carpenter training 5](#_Toc233313584)

[Group meeting 1 7](#_Toc233313585)

[Household visit 1 8](#_Toc233313586)

[Group meeting 2 8](#_Toc233313587)

[Household visit 2 10](#_Toc233313588)

[Male engagement meeting (part 1) 10](#_Toc233313589)

[Male engagement meeting (part 2) 12](#_Toc233313590)

[Group meeting 3 14](#_Toc233313591)

[Household visit 3 14](#_Toc233313592)

[Group meeting 4 16](#_Toc233313593)

[Household visit 4 16](#_Toc233313594)

[Group meeting 5 18](#_Toc233313595)

[Household visit 5 18](#_Toc233313596)

[Group meeting 6 20](#_Toc233313597)

[Household visit 6 20](#_Toc233313598)

[Fig A: Poster showing some negative consequences of keeping poultry indoors at night, shown and discussed during group meetings to encourage improved poultry and poultry feces management among households in rural Bangladesh 22](#_Toc233313599)

[Fig B: Posters showing intended behavior, shown and discussed during group meetings to encourage improved poultry and poultry feces management among households in rural Bangladesh 24](#_Toc233313600)

[Fig C: Poster showing the enabling technology of an improved poultry night shed, shown and discussed during group meetings to encourage improved poultry and poultry feces management among households in rural Bangladesh 26](#_Toc233313601)

[Fig D: Flow chart of study household selection, enrollment, and participation 28](#_Toc233313602)

[Table E: Impacts of the Neighborhood-based Environmental Assessment and Planning intervention to reduce the prevalence of poultry sleeping indoors at night and reduce the presence of poultry fecal matter in the household environment 29](#_Toc233313603)

[Table F: Impacts of the Neighborhood-based Environmental Assessment and Planning intervention to reduce the prevalence of poultry sleeping indoors at night and reduce the presence of poultry fecal matter in the household environment, by study arm 31](#_Toc233313604)

* Citation: Hopewell S, Chan A-W, Collins GS, Hróbjartsson A, Moher D, Schulz KF, et al. (2025) CONSORT 2025 statement: Updated guideline for reporting randomised trials. PLoS Med 22(4): e1004587. https://doi.org/10.1371/journal.pmed.1004587

# Table A: TiDieR-WASH Checklist

| Item | Where is this information available? |
| --- | --- |
| 1. Name | Intervention design, Intervention delivery (Pages 7-8) |
| 1. Theory of Change | Intervention design, Page 8 |
| 1. Prior evidence | Introduction, Pages 4-5; Intervention design (Pages 7-8) |
| 1. Location and setting | Setting, Page 5 |
| 1. Context | Setting; Selection of study households and participants; Intervention design; Intervention delivery (Pages 7-9) |
| 1. Suitability | Introduction, Pages 4-5 |
| 1. Implementers | Intervention design; Intervention delivery; Data collection (Pages 7-11) |
| 1. Recipients | Intervention delivery (Pages 7-10); Supplemental Information |
| 1. Targeting | Intervention delivery (Pages 7-10) |
| 1. Activities | Intervention delivery (Pages 7-10); Figure 1 |
| 1. Intervention dose | Data collection (Page 11); Supplemental Information |
| 1. Fidelity | Data collection (Page 11); Supplemental Information |
| 1. Cost | Not available |
| 1. Materials | Intervention delivery (Pages 7-10); Figure 1 |

#

# Table B: CONSORT 2010 checklist of information to include when reporting a randomised trial*

| **Section / Topic** | **No** | **CONSORT 2025 checklist item description** | **Reported on page no.** |
| --- | --- | --- | --- |
| Title and abstract | | |  |
| Title and structured abstract | 1a | Identification as a randomised trial | NA – pre-post trial |
|  | 1b | Structured summary of the trial design, methods, results, and conclusions | 2 |
| Open science | | |  |
| Trial registration | 2 | Name of trial registry, identifying number (with URL) and date of registration | NA |
| Protocol and statistical analysis plan | 3 | Where the trial protocol and statistical analysis plan can be accessed | Not available |
| Data sharing | 4 | Where and how the individual de-identified participant data (including data dictionary), statistical code and any other materials can be accessed | Statement added to the manuscript by the publisher when publishing |
| Funding and conflicts of interest | 5a | Sources of funding and other support (e.g., supply of drugs), and role of funders in the design, conduct, analysis and reporting of the trial |  |
|  | 5b | Financial and other conflicts of interest of the manuscript authors |  |
| Introduction | | |  |
| Background and rationale | 6 | Scientific background and rationale | 3-4 |
| Objectives | 7 | Specific objectives related to benefits and harms | 4 |
| Methods | | |  |
| Patient and public involvement | 8 | Details of patient or public involvement in the design, conduct and reporting of the trial | Not available |
| Trial design | 9 | Description of trial design including type of trial (e.g., parallel group, crossover), allocation ratio, and framework (e.g., superiority, equivalence, non-inferiority, exploratory) | 5 |
| Changes to trial protocol | 10 | Important changes to the trial after it commenced including any outcomes or analyses that were not prespecified, with reason | 7 |
| Trial setting | 11 | Settings (e.g., community, hospital) and locations (e.g., countries, sites) where the trial was conducted | 5 |
| Eligibility criteria | 12a | Eligibility criteria for participants | 5 |
|  | 12b | If applicable, eligibility criteria for sites and for individuals delivering the interventions (e.g., surgeons, physiotherapists) | NA |
| Intervention and comparator | 13 | Intervention and comparator with sufficient details to allow replication. If relevant, where additional materials describing the intervention and comparator (e.g., intervention manual) can be accessed | 6-7 |
| Outcomes | 14 | Pre-specified primary and secondary outcomes, including the specific measurement variable (e.g., systolic blood pressure), analysis metric (e.g., change from baseline, final value, time to event), method of aggregation (e.g., median, proportion), and time point for each outcome | 8 |
| Harms | 15 | How harms were defined and assessed (e.g., systematically, non-systematically) | NA |
| Sample size | 16a | How sample size was determined, including all assumptions supporting the sample size calculation | 6 |
|  | 16b | Explanation of any interim analyses and stopping guidelines | NA |
| Randomisation: |  |  |  |
| Sequence generation | 17a | Who generated the random allocation sequence and the method used | 5 |
|  | 17b | Type of randomisation and details of any restriction (e.g., stratification, blocking and block size) | 5 |
| Allocation concealment mechanism | 18 | Mechanism used to implement the random allocation sequence (e.g., central computer/telephone; sequentially numbered, opaque, sealed containers), describing any steps to conceal the sequence until interventions were assigned | 5 |
| Implementation | 19 | Whether the personnel who enrolled and those who assigned participants to the interventions had access to the random allocation sequence | 6 |
| Blinding | 20a | Who was blinded after assignment to interventions (e.g., participants, care providers, outcome assessors, data analysts) | 6 |
|  | 20b | If blinded, how blinding was achieved and description of the similarity of interventions | 6-7 |
| Statistical methods | 21a | Statistical methods used to compare groups for primary and secondary outcomes, including harms | 9 |
|  | 21b | Definition of who is included in each analysis (e.g., all randomised participants), and in which group | 9 |
|  | 21c | How missing data were handled in the analysis | 9 |
|  | 21d | Methods for any additional analyses (e.g., subgroup and sensitivity analyses), distinguishing prespecified from post-hoc | NA |
| Results | | |  |
| Participant flow, including flow diagram | 22a | For each group, the numbers of participants who were randomly assigned, received intended intervention, and were analysed for the primary outcome | Fig 1 |
|  | 22b | For each group, losses and exclusions after randomisation, together with reasons | 9 |
| Recruitment | 23a | Dates defining the periods of recruitment and follow-up for outcomes of benefits and harms | 9 |
|  | 23b | If relevant, why the trial ended or was stopped | 8 |
| Intervention and comparator delivery | 24a | Intervention and comparator as they were actually administered (e.g., where appropriate, who delivered the intervention/comparator, how participants adhered, whether they were delivered as intended [fidelity]) |  |
|  | 24b | Concomitant care received during the trial for each group |  |
| Baseline data | 25 | A table showing baseline demographic and clinical characteristics for each group | Table 1 |
| Numbers analysed,  outcomes and estimation | 26 | For each primary and secondary outcome, by group:   - the number of participants included in the analysis - the number of participants with available data at the outcome time point - result for each group, and the estimated effect size and its precision (such as 95% confidence interval) - for binary outcomes, presentation of both absolute and relative effect size | Page 9 and Table 1 and Table 2 |
| Harms | 27 | All harms or unintended events in each group | 9 |
| Ancillary analyses | 28 | Any other analyses performed, including subgroup and sensitivity analyses, distinguishing pre-specified from post-hoc | NA |
| Discussion | | |  |
| Interpretation | 29 | Interpretation consistent with results, balancing benefits and harms, and considering other relevant evidence | 15 |
| Limitations | 30 | Trial limitations, addressing sources of potential bias, imprecision, generalisability, and, if relevant, multiplicity of analyses | 15 |

*We strongly recommend reading this statement in conjunction with the CONSORT 2025 Explanation and Elaboration and/or the CONSORT 2025 Expanded Checklist for important clarifications on all the items. We also recommend reading relevant CONSORT extensions. See [www.consort-spirit.org](http://www.consort-spirit.org).

Citation: Hopewell S, Chan AW, Collins GS, Hróbjartsson A, Moher D, Schulz KF, et al. CONSORT 2025 Statement: updated guideline for reporting randomised trials. BMJ. 2025; 388:e081123. <https://dx.doi.org/10.1136/bmj-2024-081123>.

© 2025 Hopewell et al. This is an Open Access article distributed under the terms of the Creative Commons Attribution License (<https://creativecommons.org/licenses/by/4.0/>), which permits unrestricted use, distribution, and reproduction in any medium, provided the original work is properly cited.

# Text A: Process monitoring

As a part of process monitoring (Table S1), the CHP supervisors observed a total of 75 touchpoints with participants, including group meetings, household visits, and male engagement meetings (41 observations in the non-subsidy arm and 34 in the subsidy arm). The performance of CHPs was good or very good and similar in both study arms (Table S2). During qualitative interviews, a small number of participants mentioned feeling some pressure to build a shed due to the CHP's frequent visits. One participant said that recommendations given by CHPs may not be taken as seriously as those given by intervention staff.

# Table C: Summary of process monitoring during an intervention to encourage improved poultry and poultry feces management among households in rural Bangladesh

| **Tools** | **Objective(s)** | **Data Collectors** | **Frequency** | **Data type** |
| --- | --- | --- | --- | --- |
| Group meeting daily record form | Record the attendance, duration, and challenges of each group meeting/male engagement meeting conducted by the CHPs | CHPs | One per each group meeting with each group (primary poultry raiser/male household members) | Quantitative |
| Household visit daily record form | Record the attendance, duration, and challenges of each household visit conducted by the CHPs | Female CHPs | One per each household during each household visit with the primary poultry raisers | Quantitative |
| Pictorial progress book | Assess the progress of the households in planning and executing the recommended behavior | CHPs | One book for each household and group meeting specific targets and challenges were filled up during each household visit | Quantitative |
| Supervisor monitoring form | Record the session conduction quality and performance of the CHP | icddr,b research officers | One per CHP during group meetings; two per CHP during household visits | Quantitative |
| Observation notes (group meetings, male engagement meetings, and household visits) | Record the perspective of the icddr,b research officers regarding the meeting content, method of delivery, the interaction between the CHPs and the participants, the performance of the CHPs, and challenges | icddr,b research officers | One per each CHP during each group meeting, male engagement meeting, and household visit | Qualitative |
| Meeting-specific in-depth interview guideline for the CHPs | Record the perspective of the CHPs regarding the meeting content, method of delivery, and challenges | icddr,b research officers | One interview per CHP after completion of each group meeting (total of four after completion of each group meeting) | Qualitative |
| Meeting-specific in-depth interview guideline for the primary poultry raiser | Record the perspective of the primary poultry raiser regarding the meeting content, method of delivery, facilitator, and challenges | icddr,b research officers | One interview with one study participant from each study village (total of four after completing each group meeting) | Qualitative |
| Training notes | Record observation notes of the training facilitators regarding the training of the CHPs | Training facilitators (icddr,b research officer) | One note per training session | Qualitative |
| Training evaluation form | Record the perspective of the CHPs regarding the training modality, content, and facilitator | CHPs | Each CHP filled out one form after each training session | Quantitative & qualitative |

# Table D: Supervisor assessment of community health promoter (CHP) performance during the group meetings to encourage improved poultry and poultry feces management among households in rural Bangladesh

| Performance indicators | Non-subsidy arm (N=41)  % | Subsidy arm  (N=34)  % |
| --- | --- | --- |
| CHP provided an introduction before starting the activity | | |
| Complete | 51% | 50% |
| Partial | 42% | 44% |
| Not at all | 7% | 6% |
| CHP discussed all the key topics of the meeting | 93% | 97% |
| CHP summarized key points at the end of the meeting | 71% | 88% |
| CHP praised and encouraged making progress on action items since the previous meeting (N=60)^^[[1]](#footnote-1)^^ | 67% | 63% |
| Relationship of the CHP with the participants observed | |  |
| Good rapport | 93% | 97% |
| Mixed rapport | 7% | 3% |
| CHP's performance in demonstrating the meeting activities as perceived by the supervisor during session observation | |  |
| Very good | 73% | 59% |
| Fairly good | 22% | 41% |
| Not very good | 5% | 0% |

# Text B: Curriculum for Neighborhood-based Environmental Assessment and Planning (NEAP) approach to promote nighttime confinement of poultry outside the house and improve poultry feces management and handwashing practices

Implemented 2020-2021 in Fulbaria, Bangladesh by icddr,b

| **Session #** | **Purpose** | **Key Content** | **Materials** |
| --- | --- | --- | --- |
| Community meeting Time: 30 minutes | - Introduce project objectives and team   Generate buy-in from larger community and key stakeholders | **Project Introduction (15 minutes)**   - Provide a brief background of the project - Explain duration and procedure of project, including eligibility criteria and household selection procedures - Introduce project team members   **Demonstration and activities (15 minutes)**   - Speech on the importance of separating child from poultry and raising healthy chickens - Model-based demonstration of moving poultry from the *khachi* (bamboo basket/cage) to the night shed and pledge for participants to do the same   **Other (5 minutes)**   - Assess when men are available to come to a male engagement meeting   Snacks | - Invitation letter - Snacks - Model shed - Poultry figurines / stuffed animals - *khachi (bamboo basket/cage)*   Behavioral recommendation poster |
| Carpenter training Time: 30 minutes | - Provide local carpenters with a project overview - Train local carpenters on the characteristics of an improved shed | **Project overview (5 min)**   - Start the session with handwashing with soap/soapy water - Provide a brief about COVID-19 by showing poster - Provide a brief background of the project - Explain the role of local carpenters in the project/capacity in which they might be needed   **Discuss model shed (25 min)**   - Present the model shed to show the finished product, discuss shed construction criteria and specifications, review shed construction blueprint and discuss specific shed criteria - Distribution of shed construction blue print to use as a guide by the carpenters during constructing sheds for the households - Emphasize that households’ ideas are priority, and the sample shed is only a guideline - Provide examples of alternative shed construction needs of participating households   - Deconstruction of existing sheds to reconstruct into a new shed with recommended components   - Households continue using old shed for one type of bird and construct another shed to house other type(s) of birds - Discussion to answer any questions carpenters may have - Brief the carpenters that households will start approaching them over the next 2 weeks to engage their services - Handwashing and snacks | - Handwashing station with soapy water bottle - Handout for the masons - Shed blueprint - Model shed - Snacks |
| Group meeting 1 Time: 40 minutes | - Provide participating poultry-raisers with an overview of benefits/challenges of poultry raising - Introduce child health risks related to poultry raising - Introduce key behavioral recommendations - Pledge to participate | **Introductions (5 min)**   - Start the session with handwashing with soap/soapy water - Have the participants sit in a circle - Ask who attended community meeting - Ask one participant what they remember from community meeting - Preventive behavioral recommendations against COVID-19   **Benefits/challenges of poultry raising (10 min)**   - Use Flipbook to facilitate discussion about benefits and challenges of raising poultry   **Introduce intervention content; pledge to participate (15 min)**   - Show Key Behavioral Recommendations Poster - Describe the behavioral recommendations / key messages on one poster - Ask if participants have their own aspirations related to poultry raising, project behavioral recommendations   - What is the main thing they would like to accomplish through this project? - Distribute small poster with image of 4 behavioral recommendations - Have participants take an oral pledge to participate in the sessions and practice the behavioral recommendations. - Ask participants to put a thumbprint on/sign their poster to signal their pledge   **Closing (5 min)**   - End session by detailing expectations for participants, including attendance and decide on time for next meeting   **Action Items (5 min)**   - Hang/Display poster in household - Share with household members what you have learned in the session and the commitment that you have made | - Handwashing station with soapy water bottle - Mat to sit on - Session guide - Flipbook - Key Behavioral Recommendations Poster - Printed Key behavioral Recommendations Posters for households - Script with pledge to be read aloud - Ink pad/pen |
| Household visit 1 Time: 20 minutes | - CHPs discuss household comments and concerns | **Activities**   - Comments and concerns   - What did you understand from the first session, what did you find difficult? - CHPs check that Key Recommendations Poster has been put up in the house   **Action items for CHPs**   - Complete CHP pictorial progress book | - FAQ script - Pictorial progress book |
| Group meeting 2 Time: 50 minutes | - Discuss negative consequences of confining poultry inside the household at night - Discuss benefits of confining poultry outside the living space at night - Brief overview of and instructions for constructing a night shed | **Introductions (5 min)**   - Greet participants - Start the session with handwashing with soap/soapy water   - Poultry figurine and model shed next to handwashing station for ritual   **Behavioral Recommendation 1: Confine all poultry out of the living space at night (15 min)**   - Discuss BR1 NEAP Posters - Discuss negative consequences of confining poultry inside the household at night - Discuss benefits of confining poultry outside the living space at night - Introduce technology (model shed)   **Play the videos (10 minutes)**   - Show videos on benefits of confining poultry in a shed at night (e.g. reduction of feces and smell inside the house) - The video contains:   - Benefits of poultry housing out of living space   - Explain shed characteristics   **Introduce subsidy (subsidy arm only)**   - This is not full support - They need to have collected some materials or communicated with mason to receive the money   **Review next steps (5 minutes)**   - Explain next steps   - Male engagement meeting   - CHP visits   **Action item (5 minutes)**   - Share and discuss BR1 with other household members - Share and discuss shed construction with other household members, especially those with financial and/or decision-making power - Discuss what materials are on hand that can be used for shed construction - Make a list of materials you have | - Model shed - Handwashing station - Soapy water bottle - Poultry doll / figurine - NEAP posters (BR1) - Sticks to use as pointers - Videos - Tablets to show videos - Pictorial progress book - Mat to sit on - Session guide |
| Household visit 2 Time: 20 minutes | - CHPs work with households to assess conditions and resources in preparation for shed construction - CHPs discuss household comments and concerns - Introduce participant pictorial progress book - CHPs work with households to complete pictorial progress book | **Activities**   - Check that Key Recommendations Poster is still intact and visible in house - CHPs work with households to complete household visit-2 pictorial progress book page - Night shed: at night, confine all the poultry outside the living space - CHPs discuss challenges and concerns with households   **Action items for CHPs**   - Complete pictorial progress book | - Pictorial progress book - FAQ script |
| Male engagement meeting (part 1) Time: 50 minutes | - Provide male household members with an overview of the project and their expected contributions - Provide male household members with an overview of benefits/challenges of poultry raising - Introduce child health risks related to poultry raising - Introduce key behavioral recommendations | **Introductions (10 min)**   - Start the session with handwashing with soap/soapy water - Ask who attended community meeting - Ask one participant what they remember from community meeting - Preventive behavioral recommendations against COVID-19   **Introduce intervention content; pledge to participate (15 min)**   - Use a short version of flipbook to facilitate the discussion about benefits of raising poultry and consequences of keeping poultry inside the living space at night - Show Key Behavioral Recommendations poster and describe it - Explain the role of male household members in the project/capacity in which they might contribute (card sorting exercise) - Brief overview of the project, why men should be involved, and their contribution to the health and safety of the family (story telling of two fathers) - Note that most households in the intervention will NOT have a child <5 at the house so we need to focus on poultry raising and benefits to health of children in the COMPOUND   **Action Items (5 min)**   - Share with household members what you have learned in the session - Make a plan for a night shed, consulting with poultry-raiser - Support the poultry-raiser to remove poultry out of the living space at night, support her for building a shed with money, materials and labor - Work together as a household to ensure child health and poultry health | - Handwashing station - Soapy water bottle - Mat to sit on - Session guide - Key Behavioral Recommendations Poster - Flipbook - Posters of preventive behavioral recommendations against COVID-19 - Activity listed cards for card sorting exercise - Stick for pointing BR poster - Man/woman/both image for card sorting activity - Lights/lanterns (if needed) |
| Male engagement meeting (part 2) Time: 1 hour | - Discuss negative consequences of confining poultry inside the household at night - Discuss benefits of confining poultry outside the living space at night - Brief overview of and instructions for constructing a night shed | **Introductions (10 min)**   - Start the session with handwashing with soap/soapy water - Note that most household in the intervention will NOT have a child <5 at the house so we need to focus on poultry-raising and benefits to health of children in the COMPOUND - For subsidy villages: Mention subsidy. - For non subsidy villages: Save money by identifying what they can do - Build or repair or renovate   **Behavioral Recommendations 1 (15 minutes)**   - Discuss BR1 NEAP posters - Discuss negative consequences of confining poultry inside the household at night. - Discuss benefits of confining poultry outside the living space at night. - Introduce technology (model shed)   **Play the videos (10 minutes)**   - The video contains:   - Benefits of poultry raising out of living space   - Explain shed characteristics   **Introduce subsidy (subsidy arm only).**   - This is not full support - They need to have collected some materials or communicated with mason to receive the money   **Model shed (15 minutes)**   - Answer participant questions; discussion of the sessions to address any potential difficulties and corresponding solutions   **Review next steps (5 minutes)**   - Explain next steps - CHP visits - Notes on progress and difficulties   **Action items (5 minutes)**   - Determine the amount of money you have for a shed - Work with your other household members to decide on where to put the shed - Discuss what materials have on hand that can be used for shed construction - Make a list of materials you have - Work with the primary poultry-raiser on design and shed needs - Initiate discussion with your mason to come up with a timeline for construction | - NEAP posters BR1 - Sticks to use as pointers - Model shed - Videos - Tablets to show video - Mat to sit on - Session guide - Handwashing station - Soapy water bottle |
| Group meeting 3 Time: 40 minutes | - Discuss importance of maintaining a healthy environment for poultry health - Discuss importance of maintaining a healthy environment for child health | **Introductions (5 min)**   - Greet participants. - Start the session with handwashing with soap/soapy water   - Poultry figurine and model shed next to handwashing station for ritual.   **Behavioral Recommendation 3: Remove poultry feces as soon as you see them (10 minutes)**   - Discuss BR3 NEAP Poster - Discuss negative consequences of not cleaning feces as soon as you see them - Discuss negative consequences of not cleaning feces from the poultry shed. - Discuss benefits of cleaning feces as soon as you see them - Discuss benefits of cleaning feces from the poultry shed - Discuss importance of doing these things for poultry health - Discuss importance of doing these things for child health   **Handwashing Primer (2-3 minutes)**   - Remind households that hands should be washed with soap/soapy water after cleaning any feces.   **Poultry Health Tip #1 (2-3 minutes)**   - Provide households with a laying hens’ health tip   **Poultry shed construction troubleshooting (15 minutes)**   - Discuss successes and difficulties on progress of shed construction.   **Action items (2-3 minutes)**   - Get a designated *cheni*/hoe/spade for feces disposal | - Model shed - Handwashing station - Soapy water bottle - Poultry doll / figurine - NEAP posters BR3 - Pointer sticks - Mat to sit on - Session guide |
| Household visit 3 Time: 20 minutes | - CHPs assess progress of night shed construction - CHPs assess progress of obtaining feces disposal tool - CHPs work with households to complete pictorial progress book | **Activities**   - CHPs review household visit-3 pictorial progress book page - Night shed: at night, confine all the poultry outside the living space - Feces removal and disposal: Remove poultry feces as soon as you see them and dispose of them in a designated location - CHPs discuss challenges and concerns with households   **Action items for CHPs**   - Complete pictorial progress book - Help participants prepare for feces disposal session   - Walk with participants around compound to look for suitable place for disposing poultry feces, at a suitable distance away from children play area. Recommend behind latrine, if possible. | - Pictorial progress book - FAQ script |
| Group meeting 4 Time: 30 minutes | - Discuss benefits of disposing poultry feces in a specific place - Discuss importance of maintaining a healthy environment for child health - Discuss importance of maintaining a healthy environment for human health | **Introductions (5 min)**   - Greet participants. - Start the session with handwashing with soap/soapy water   - Poultry figurine and model shed next to handwashing station for ritual. - Review of cleaning poultry feces (group session 3)   **Behavioral Recommendation 4: Dispose of poultry feces in a specific place (15 minutes)**   - Introduce the importance of disposing poultry feces safely   - The benefits of disposing poultry feces in a specific place for children - Behind the latrine - Far from courtyard/living space/home - A pit where other animal feces are disposed - Or any other place that is away from children and household   - The negative consequence of disposing poultry feces here and there/pond for the environment and human health   **Handwashing Primer (2-3 minutes)**   - Remind households that hands should be washed with soap/soapy water after cleaning any feces.   **Poultry Health Tip #2 (2-3 minutes)**   - Provide households with a hatching hens’ health tip   **Action items (5 minutes)**   - Identify a location for disposing poultry feces - Promise to start disposing both big and small piles of poultry feces in that place | - Model shed - Handwashing station - Soapy water bottle - Poultry doll / figurine - NEAP posters BR4 - Pointer sticks - Mat to sit on - Session guide |
| Household visit 4 Time: 20 minutes | - CHPs assess progress of night shed construction - CHPs assess progress of obtaining feces disposal tool - CHPs assess the identification and use of a specific place for poultry feces disposal which is out of children’s reach - CHPs work with households to complete pictorial progress book | **Activities**   - CHPs review household visit- 4 pictorial progress book page - Night shed: at night, confine all the poultry outside the living space - Feces removal and disposal: Remove poultry feces as soon as you see them and dispose of them in a designated location - Handwashing: wash hands with soap and water after contact with poultry, poultry products or poultry feces - CHPs discuss challenges and concerns with households   **Action items for CHPs**   - Complete pictorial progress book | - Pictorial progress book - FAQ script |
| Group meeting 5 Time: 40 minutes | - Discuss importance of handwashing at key times - Discuss vaccines as a method of keeping poultry healthy | **Introductions (5 min)**   - Greet participants. - Start the session with handwashing with soap/soapy water   - Poultry figurine and model shed next to handwashing station for ritual   **Behavioral Recommendation 2: Wash hands with soap and water after contact with poultry, poultry products, or poultry feces (10 minutes)**   - Discuss negative consequences of poor hand hygiene - Discuss benefits of good hand hygiene - Introduce key times to wash hands related to poultry and general health - Discuss benefits of hand washing with soap and water after contact with poultry, poultry products, or poultry feces   **Demonstration (10 minutes)**   - Facilitator demonstrates and instructs on handwashing station construction - Facilitator demonstrates and instructs on soapy water making - Group discussion on alternative sources of handwashing or activity on making soapy water bottles.   **Poultry Health Tip #3 (2-3 minutes)**   - Provide households with a poultry health tip related to maintaining chicks’ health and feed - **Action items:** Make 1-2 soapy water bottles to be placed at tube-well and near food preparation and feeding area. | - Model shed - Handwashing station - Soapy water bottle - Poultry doll / figurine - NEAP poster BR2 - Pointer sticks - Session guide - Mat to sit on - Detergent - Empty bottle |
| Household visit 5 Time: 20 minutes | - CHPs assess progress of night shed construction - CHPs assess progress of obtaining feces disposal tool - CHPs assess the use of a specific place for poultry feces disposal which is out of children’s reach - CHPs assess progress of handwashing recommendations - CHPs work with households to complete pictorial progress book | **Activities**   - CHPs review household visit- 5 pictorial progress book page - Night shed: at night, confine all the poultry outside the living space - Feces removal and disposal: Remove poultry feces as soon as you see them and dispose of them in a designated location - Handwashing: wash hands with soap and water after contact with poultry, poultry products or poultry feces - CHPs discuss challenges and concerns with households   **Action items for CHPs**   - Complete pictorial progress book | - Pictorial progress book - FAQ form |
| Group meeting 6 Time: 30 minutes | - Discuss successes and difficulties on progress of shed construction - Discuss successes and difficulties with keeping all poultry out of the sleeping space at night - Discuss successes and difficulties on feces removal as soon as possible and disposing in a specific place - Remind households about key times of handwashing - Review of past 12 weeks and lessons learned | **Introductions (5 min)**   - Greet participants. - Start the session with handwashing with soap/soapy water   - Poultry figurine and model shed next to handwashing station for ritual.   **Activity (15 min)**   - Discuss successes and difficulties on progress of shed construction. Address any issues (5 min) - Review of past 12 weeks and lessons learned (5 min)   - Ask 2-3 participants what they have learned in past 12 weeks and add if necessary - Remind participants of the key behavioral recommendations and summarize the benefits of key behavioral recommendations of the project - Discussion on phase out recommendation (5 min)   - Remind the participants to continue practicing the key behavioral recommendations   **Handwashing Primer (2-3 minutes)**   - Remind households that hands should be washed after cleaning any feces.   **Poultry Health Tip #4 (2-3 minutes)**   - Provide households with a poultry health tip related to treatment of chicken and chicks | - Model shed - Handwashing station - Soapy water bottle - Poultry doll / figurine - Session guide - Mat to sit on - Poster of key behavioral recommendation |
| Household visit 6 Time: 20 minutes | - CHPs assess progress of night shed construction - CHPs assess progress of obtaining feces disposal tool - CHPs assess progress of handwashing recommendations - CHPs work with households to complete pictorial progress book | **Activities**   - CHPs review household visit- 4 pictorial progress book page - Night shed: at night, confine all the poultry outside the living space - Feces removal and disposal: Remove poultry feces as soon as you see them and dispose of them in a designated location - Handwashing: wash hands with soap and water after contact with poultry, poultry products or poultry feces - CHPs discuss challenges and concerns with households   **Action items for CHPs**   - Complete pictorial progress book | - FAQ form - Pictorial progress book |

# Fig A: Poster showing some negative consequences of keeping poultry indoors at night, shown and discussed during group meetings to encourage improved poultry and poultry feces management among households in rural Bangladesh

Each panel was used to explain a negative consequence of the common behavior of housing poultry indoors. Following a discussion of the poster, the facilitator of the group session asked participants how they could move from the common behaviors described to the recommended behaviors.

Panel 1 shows a picture of a sleeping room, where a mother and a child are sleeping and poultry are kept under the bed. It was used to prompt a discussion about negative health impacts that are more likely when children and poultry sleep in the same room and the negative impacts on poultry from poor ventilation.

Panel 2 shows poultry feathers and feces next to the poultry cages by the bed and the child trying to interact with the poultry. It was used to emphasize that children can easily come into contact with poultry and poultry feces when poultry are kept in the house.

Panel 3 shows a guest holding her nose because of the smell resulting from the poultry and the ashamed homeowner. The image prompted a discussion about how the situation might be different if the mother did not keep poultry in the room.

Panel 4 shows a snake approaching poultry that have been confined under the bed. The image was used to explain how confining poultry under the bed can negatively affect poultry health due to predation, a lack of ventilation, and a lack of space to move around.

Panel 5 shows a family with free-ranging poultry that have defecated within the domestic space, potentially causing the child to fall ill. The image was used to discuss the pathogens that are carried by poultry and how they can result in infections that may or may not be symptomatic but can still be harmful.


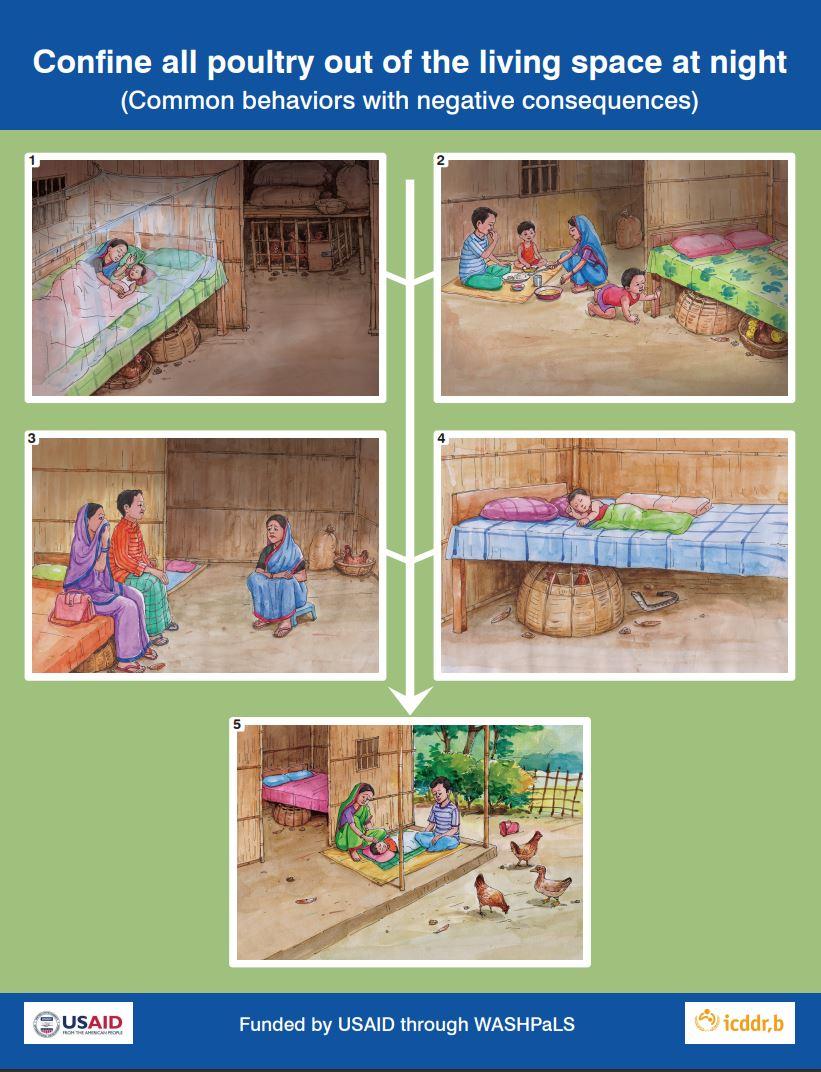


# Fig B: Posters showing intended behavior, shown and discussed during group meetings to encourage improved poultry and poultry feces management among households in rural Bangladesh

Each panel was used to explain the benefits of not housing poultry indoors. Following a discussion of the poster, the facilitator of the group session explained to participants that they could keep poultry outside of their houses by constructing and using an improved poultry shed.

Panel 1 shows a child playing with his grandmother in a room that has no sign of poultry. The discussion facilitator emphasized how the clean environment was good for child growth and health.

Panel 2 shows a guest visiting a room that has no sign of poultry. The image was used to discuss how the mother felt much more comfortable hosting guests in a clean house than a room that housed poultry.

Panel 3 shows an improved poultry shed. The image was used to discuss how a poultry shed can provide poultry with sufficient ventilation, space, and light.

Panel 4 shows a man keeping chicks in one compartment of the shed and a laying hen in another compartment. The image was used to explain that an improved poultry shed with multiple compartments can be used to house poultry at all stages so that no poultry need to sleep inside the house.

Panel 5 shows a family welcoming a child home from school. There are no signs of poultry or poultry feces. The group facilitator used the image to emphasize how a clean environment supports the health of children.


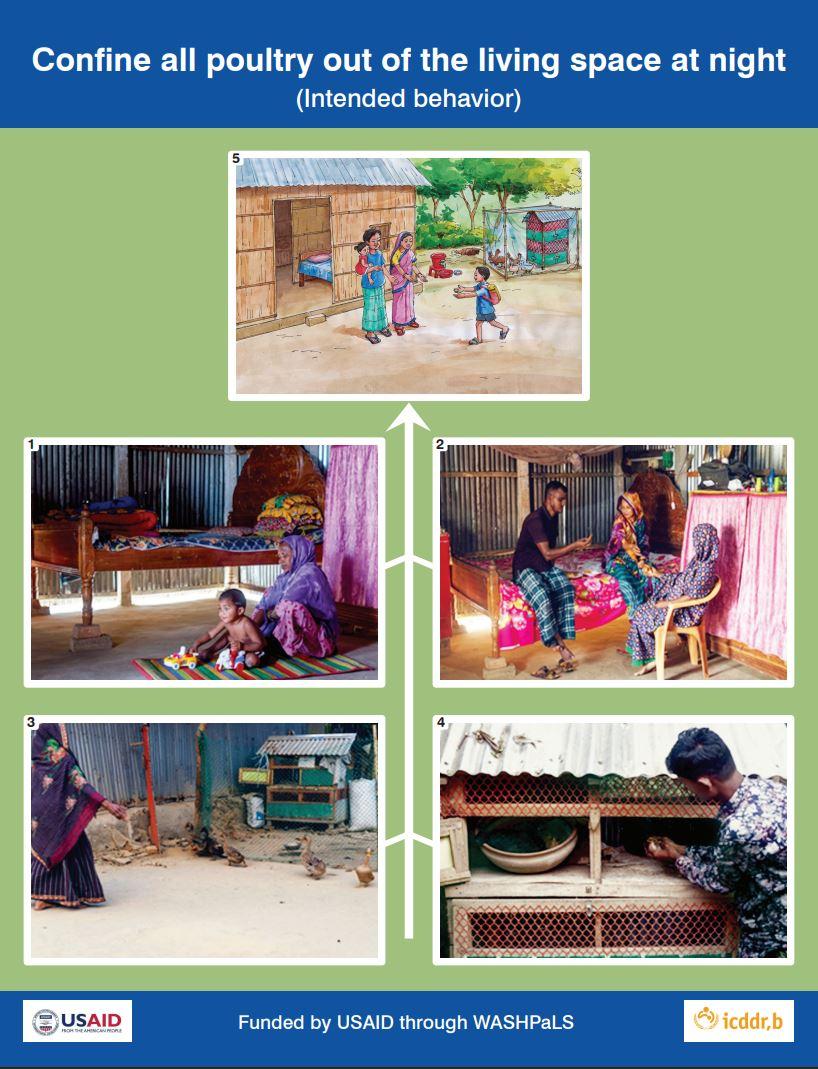


# Fig C: Poster showing the enabling technology of an improved poultry night shed, shown and discussed during group meetings to encourage improved poultry and poultry feces management among households in rural Bangladesh


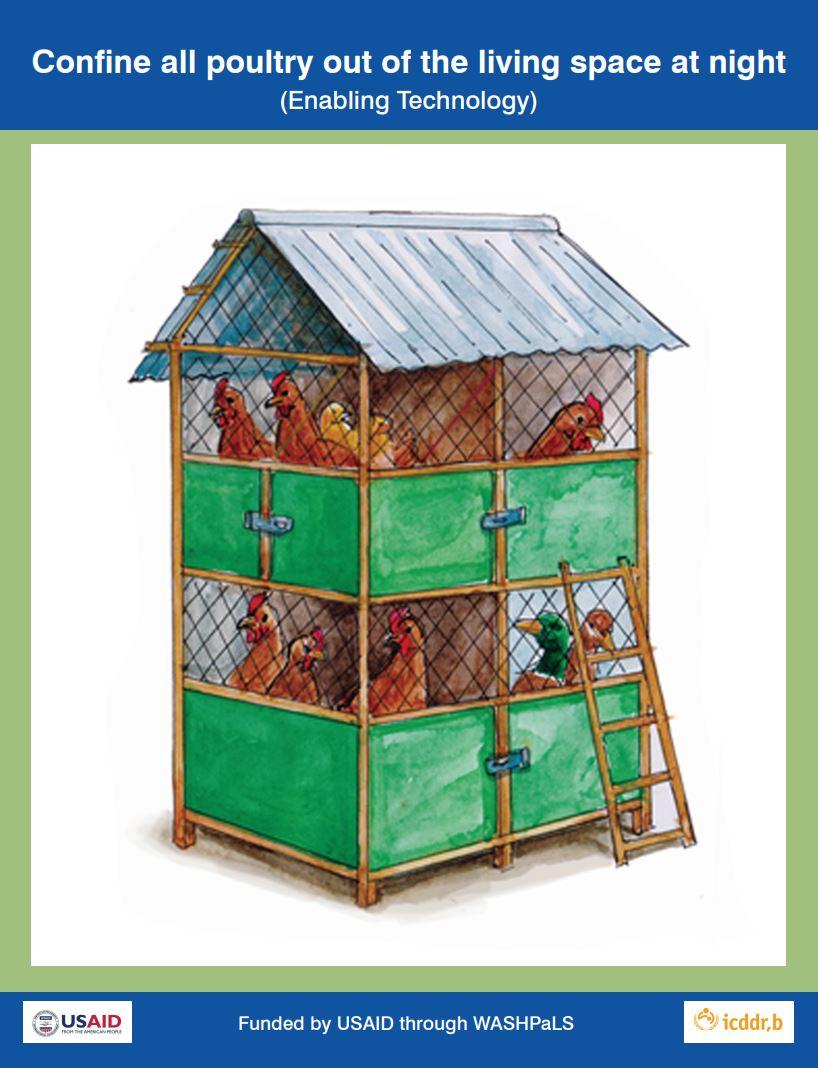


Fig D: Flow chart of study household selection, enrollment, and participation


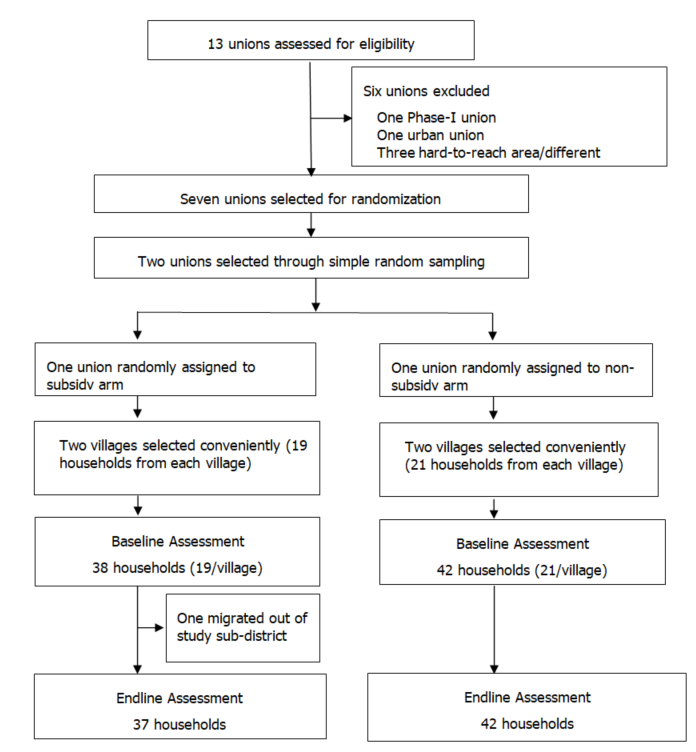


# Table E: Impacts of the Neighborhood-based Environmental Assessment and Planning intervention to reduce the prevalence of poultry sleeping indoors at night and reduce the presence of poultry fecal matter in the household environment

| **Indicators** | **Baseline**  **(n = 79)**  **n (%)** | **Endline**  **(n = 79)**  **n (%)** | **Prevalence difference (baseline-** **to-** **endline)**  **pp [95% CI]** |
| --- | --- | --- | --- |
| Percent of respondents reported seeing uncontained poultry feces in the household dwelling | n = 79 | n = 79 |  |
| Never | 5 (6.3%) | 7 (8.9%) | 3 [-6, 11] |
| **Within one week** | 70 (89%) | 57 (72%) | **-16 [-27, -6]** |
| Percent of respondents reported seeing their <5 child play with or grabbed poultry | n = 42 | n = 44 |  |
| Never | 11 (26%) | 9 (21%) | -10 [-25, 6] |
| Within one week | 21 (50%) | 15 (34%) | -14 [-37, 8] |
| Percent of respondents reported seeing <5 child put poultry feces in their mouth | n = 42 | n = 44 |  |
| Never | 26 (62%) | 29 (66%) | 2 [-14, 19] |
| Within one week | 1 (2%) | 0 (0%) | 0 [-7, 7] |
| Percent of respondents reported seeing <5 child touch poultry feces | n = 42 | n = 44 |  |
| Never | 9 (21%) | 14 (32%) | 7 [-9, 34] |
| **Within one week** | 13 (31%) | 4 (9%) | **-21 [-38, -5]** |
| Percent of respondents reported seeing the <5 child step on poultry feces | n = 42 | n = 44 |  |
| Never | 4 (9.5%) | 7 (16%) | 5 [-11, 20] |
| Within one week | 30 (71%) | 27 (61%) | -10 [-27, 8] |
| Percent of respondents reported seeing poultry enter their <5 child's sleeping room | n = 42 | n = 44 |  |
| Never | 3 (7%) | 2 (5%) | -2 [-13, 8] |
| Within one week | 37 (88%) | 37 (84%) | -5 [-22, 12] |
| Percent of respondents reported seeing their <5 child enter the shed or other poultry confinement strategies | n = 42 | n = 44 |  |
| Never | 12 (29%) | 16 (36%) | 5 [-13, 23] |
| **Within one week** | 23 (55%) | 10 (23%) | **-31 [-52, -10]** |
| Percent of respondents reported seeing poultry eating food from the main serving dish or person's plate | n = 79 | n = 79 |  |
| Never | 7 (8.9%) | 12 (15%) | 6 [-2, 15] |
| Within one week | 42 (53%) | 34 (43%) | -10 [-25, 4] |

Bolded values indicate those that are significantly different between timepoints

^a^ An outdoor, multi-compartment poultry night-shed with cross-ventilation that is elevated off the ground

^b^ Three households had access to an improved shed that were owned by other households of the same compound

^c^During endline enumerators could not access the courtyards of two non-subsidy households to observe and count the poultry feces

# Table F: Impacts of the Neighborhood-based Environmental Assessment and Planning intervention to reduce the prevalence of poultry sleeping indoors at night and reduce the presence of poultry fecal matter in the household environment, by study arm

| Indicators | Baseline  n (%) | | Endline  n (%) | | Prevalence Difference  (baseline- to- endline) | | Effect size (difference-in-differences)  pp (95% CI) |
| --- | --- | --- | --- | --- | --- | --- | --- |
|  | Non-subsidy | Subsidy | Non-subsidy | Subsidy | Non-subsidy  pp (95% CI) | Subsidy  pp (95% CI) |  |
| Percent of respondents reported seeing uncontained poultry feces in the household dwelling | n = 42 | n = 37 | n = 42 | n = 37 | n = 42 | n = 37 |  |
| Never | 2 (5%) | 3 (8%) | 4 (10%) | 3 (8%) | 5 [-7, 17] | 0 [-14, 14] | -5 [-22, 13] |
| Within one week | 36 (86%) | 34 (92%) | 33 (79%) | 24 (65%) | -7 [-18, 4] | **-27 [-46, -8]** | -20 [-40, 1] |
| Percent of respondents reported seeing their <5 child play with or grabbed poultry | n = 21 | n = 21 | n = 22 | n = 22 | n = 21 | n = 21 |  |
| Never | 9 (43%) | 2 (10%) | 6 (27%) | 3 (14%) | -19 [-42, 4] | 0 [-20, 20] | 19 [-11, 49] |
| Within one week | 8 (38%) | 13 (62%) | 4 (18%) | 11 (50%) | -19 [-50, 12] | -10 [-45, 25] | 10 [-36, 55] |
| Percent of respondents reported seeing <5 child put poultry feces in their mouth | n = 21 | n = 21 | n = 22 | n = 22 | n = 21 | n = 21 |  |
| Never | 16 (76%) | 10 (48%) | 16 (72%) | 13 (59%) | -5 [-27, 18] | 10 [-15, 34] | 14 [-18, 47] |
| Within one week | 0 (0%) | 1 (4.8%) | 0 (0%) | 0 (0%) | 5 [-5, 15] | -5 [-15, 5] | -10 [-23, 4] |
| Percent of respondents reported seeing <5 child touch poultry feces | n = 21 | n = 21 | n = 22 | n = 22 | n = 21 | n = 21 |  |
| Never | 4 (19%) | 5 (24%) | 5 (23%) | 9 (41%) | 0 [-20, 20] | 14 [-12, 40] | 14 [-18, 46] |
| Within one week | 7 (33%) | 6 (29%) | 3 (14%) | 1 (4.6%) | -19 [-42, 4] | -24 [-48, 1] | -5 [-38, 28] |
| Percent of respondents reported seeing the <5 child step on poultry feces | n =21 | n = 21 | n = 22 | n = 22 | n = 21 | n = 21 |  |
| Never | 3 (14%) | 1 (4.8%) | 4 (18%) | 3 (14%) | 0 [-25, 25] | 10 [-10, 29] | 10 [-21, 40] |
| Within one week | 16 (76%) | 14 (67%) | 13 (59%) | 14 (64%) | -14 [-40, 12] | -5 [-32, 22] | 10 [-27, 46] |
| Percent of respondents reported seeing poultry enter their <5 child's sleeping room | n = 21 | n = 21 | n = 22 | n = 22 | n = 21 | n = 21 |  |
| Never | 2 (9.5%) | 1 (4.8%) | 1 (4.6%) | 1 (4.6%) | -5 [-22, 13] | 0 [-14, 14] | 5 [-17, 27] |
| Within one week | 18 (86%) | 19 (90%) | 19 (86%) | 18 (82%) | 0 [-25, 25] | -10 [-34, 15] | -10 [-43, 24] |
| Percent of respondents reported seeing their <5 child enter the shed or other poultry confinement strategies | n = 21 | n = 21 | n = 22 | n = 22 | n = 21 | n = 21 |  |
| Never | 7 (33%) | 5 (24%) | 8 (36%) | 8 (36%) | 0 [-25, 25] | 10 [-19, 38] | 10 [-27, 46] |
| Within one week | 11 (52%) | 12 (57%) | 4 (18%) | 6 (27%) | **-33 [-60, -7]** | -29 [-64, 7] | 5 [-38, 48] |
| Percent of respondents reported seeing poultry eating food from the main serving dish or person's plate | n = 42 | n = 37 | n = 42 | n = 37 | n = 42 | n = 37 |  |
| Never | 5 (12%) | 2 (5%) | 7 (17%) | 5 (14%) | 5 [-9, 18] | 8 [-1, 17] | 6 [-13, 20] |
| Within one week | 20 (48%) | 22 (59%) | 17 (40%) | 17 (46%) | -7 [-28, 14] | -14 [-35, 8] | -6 [-36, 23] |
| Bolded values indicate those that are significantly different between groups across timepoints  ^a^ An outdoor, multi-compartment poultry night-shed with cross-ventilation that is elevated off the ground  ^b^ Three households had access to an improved shed that were owned by another household in the same compound  ^c^ During endline enumerators could not access the courtyards of two non-subsidy households to observe and count the poultry feces | | | | | | | |

1. Not applicable for the first group meeting, household visit, and male engagement meeting. Thus here the “N” is lower than the total observed session [↑](#footnote-ref-1)
